# Rab7 is required for mesoderm patterning and gastrulation in *Xenopus*

**DOI:** 10.1242/bio.056887

**Published:** 2021-07-14

**Authors:** Jennifer Kreis, Fee M. Wielath, Philipp Vick

**Affiliations:** Department of Zoology, Institute of Biology, University of Hohenheim, 70599 Stuttgart, Germany

**Keywords:** Rab7, Endosomes, Wnt, Mesoderm, Gastrulation, *Xenopus*

## Abstract

Early embryogenesis requires tightly controlled temporal and spatial coordination of cellular behavior and signaling. Modulations are achieved at multiple levels, from cellular transcription to tissue-scale behavior. Intracellularly, the endolysosomal system emerges as an important regulator at different levels, but *in vivo* studies are rare. In the frog *Xenopus*, little is known about the developmental roles of endosomal regulators, or their potential involvement in signaling, especially for late endosomes. Here, we analyzed a hypothesized role of Rab7 in this context, a small GTPase known for its role as a late endosomal regulator. First, *rab7* showed strong maternal expression. Following localized zygotic transcript enrichment in the mesodermal ring and neural plate, it was found in tailbud-stage neural ectoderm, notochord, pronephros, eyes and neural crest tissues. Inhibition resulted in strong axis defects caused by a requirement of *rab7* for mesodermal patterning and correct gastrulation movements. To test a potential involvement in growth factor signaling, we analyzed early Wnt-dependent processes in the mesoderm. Our results suggest a selective requirement for ligand-induced Wnt activation, implicating a context-dependent role of Rab7.

## Abstract (precis)

**Summary:** The late endosomal regulator Rab7 is required for gastrulation movements and axis elongation in *Xenopus* by regulating early mesoderm patterning.

## INTRODUCTION

Early embryonic processes like germ layer formation, induction of body axes, gastrulation, neural induction and tissue differentiation require tight control of cellular processes, including temporal and spatial activation of specific sets of signaling pathways. Regulation of endocytosis or membrane trafficking can control activation, intensity, or duration of signal transduction following receptor activation (Sigismund et al., 2012). However, this has only been analyzed in few developmental processes *in vivo*, as altering basic cellular processes can have dramatic effects.

Endocytosis of membrane receptors is considered a way of downregulation of signaling. Receptors are translocated to early endosomes (EE), which represent first intracellular sorting platforms (Platta and Stenmark, 2011). From here, receptor complexes can be inactivated and recycled back to the plasma membrane. Alternatively, they are retained in EE membranes while these organelles mature into late endosomes (LE). There, transmembrane cargo can be translocated into intraluminal vesicles (ILV) by successive inward budding of the limiting membrane, a process characteristic for maturing LE, and which is performed by the endosomal sorting complexes required for transport (ESCRT) machinery (Hanson and Cashikar, 2012). Any cargo translocated into ILV is destined for acidic degradation, as LE fuse with lysosomes. Thus classically, LE represent an intermediate step between EE and degradation (Dobrowolski and De Robertis, 2011; Horner et al., 2018; Katzmann et al., 2002).

The role of endocytosis and endosomes for activation of signaling are much less understood. Several pathways require endocytosis for activation, or further transport to EE, where activating adapters are localized (Brunt and Scholpp, 2018; Butler and Wallingford, 2017; Dobrowolski and De Robertis, 2011; Fürthauer and González-Gaitán, 2009; Platta and Stenmark, 2011). A much rarer case is a positive role of LE for pathway activation, which has been suggested for epithelial growth factor (EGF) receptor-mediated mitogen-activated protein kinase (MAPK) activation (Platta and Stenmark, 2011; Teis et al., 2002). Further, for canonical Wnt signaling (from here on simply ‘Wnt signaling’), it has been demonstrated that LE are indispensable for maintaining intracellular signaling after endocytosis-mediated activation of the receptor complex (Platta and Stenmark, 2011; Taelman et al., 2010; Vinyoles et al., 2014).

Rab family proteins are a group of small GTPases that regulate membrane trafficking processes by transiently binding membranes and serving as process-specific molecular switches. Each Rab attaches to certain types of membrane or organelle and orchestrates recruitment of a specific set of effectors, thereby giving membranes an ‘identity’ and function (Stenmark, 2009). As judged by their general roles in cellular transport, many Rab proteins are categorized as ‘housekeeping genes’ (Hounkpe et al., 2021). However, they might be involved in tissue-specific processes requiring membrane transport.

The LE regulator Rab7a (from here on ‘Rab7’) and its low-expressed, tissue-specific paralog Rab7b are mainly found on LE. Thus, Rab7 is used as an LE marker, and, as a recruiter of many effectors, it is the main regulator of LE maturation and function (Huotari and Helenius, 2011; Stenmark, 2009). Concerning signaling pathways, it would thus be considered to be a permissive regulator of endolysosomal degradation, i.e. required for receptor degradation and termination of signaling (Platta and Stenmark, 2011). While this is straightforward logic, controlling activity of Rab7 could be a way of positively regulating downstream signal transducers as well. For instance, this might be the case for Wnt signaling, where functional LE have been shown to be required for sustained activation. In addition, the Wnt pathway has been reported to influence expression of endosomal regulators in a positive feedback loop, i.e. directly regulating Rab7 activity (Ploper and De Robertis, 2015; Ploper et al., 2015; Taelman et al., 2010).

Studies dealing with the *in vivo* function of Rab7 are rare, especially in a developmental context. Most information about its influence on cellular function derives from work in cell culture, i.e. from out-of-tissue contexts (Guerra and Bucci, 2016). This might be due to the general cellular role of Rab7, causing classical knockout (KO) approaches to result in embryonic lethality, as exemplified by work in the mouse. KO embryos had strong defects in endosomal transport in the anterior visceral endoderm (AVE), which resulted in antero-posterior (AP) patterning defects, and thus in failure to complete gastrulation (Kawamura et al., 2012). In a recent follow-up report, the authors further demonstrated that these phenotypes correlated with reduced Wnt signaling in the mesoderm, resulting in impaired mesoderm patterning (Kawamura et al., 2020).

In this work, we analyzed the *in vivo* function of *rab7* during *Xenopus* early embryogenesis, including a potential participation in Wnt pathway activation. In contrast to an expected general housekeeping role in all tissues, we found *rab7* mRNA specifically enriched in distinct types of tissues, reflecting dynamic changes of enhanced requirement. Using morpholino-mediated knockdown and CRISPR/Cas9-mediated genome editing, we found that loss of *rab7* specifically resulted in gastrulation defects without impacting embryonic organizer induction. Furthermore, *rab7* was required endogenously for expression of Wnt-dependent genes in the dorsal and ventral mesoderm, as well as for ligand-mediated activation of exogenously induced Wnt signaling.

## RESULTS

### Loss of *rab7* results in gastrulation defects

**Fig. 1.**
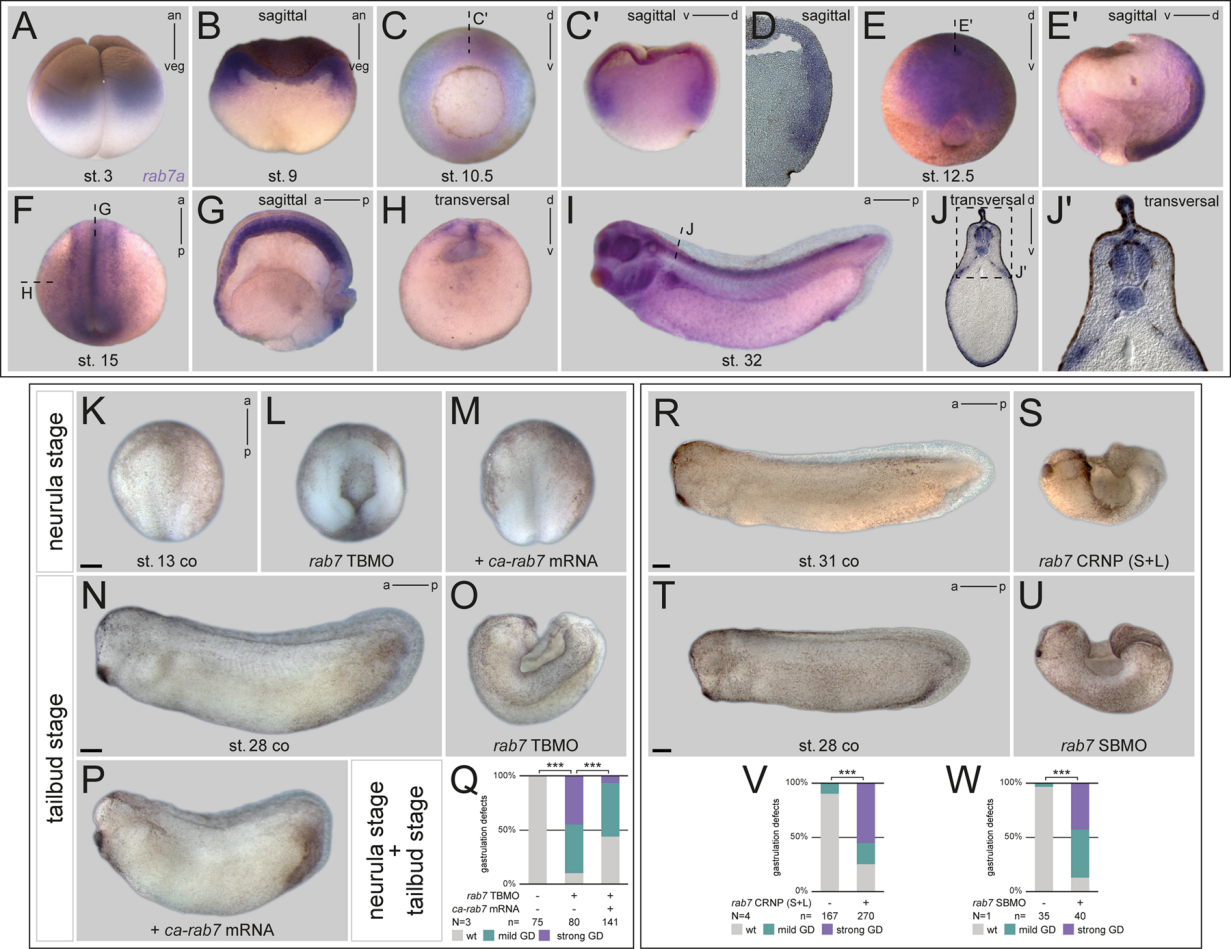
***rab7* shows dynamic expression and inhibition resulted in gastrulation defects.** (A,B) Expression of *rab7* mRNA in animal hemisphere at stage 3 and sagittal section of late blastula stage. (C) Upon gastrulation transcripts were enriched in deep mesodermal ring, (C′) sagittal section as indicated in (C), (D) dorsal area close-up view of a vibratome-sectioned specimen. (E) At stage 12.5 *rab7* accumulates in neural plate ectoderm, (E′) sagittal section of E. (F) During neurulation transcripts get restricted to notochord, neural tube and brain tissue, (G) sagittal and (H) transversal section indicated in F. (I) In tailbud stages transcripts were detected in the notochord and pronephric and head tissues, (J) transversal section indicated in I, (J′) enlargement of J. (K,N) Control specimen at stages 13 and 28. (L,O) Injection of *rab7* TBMO in dorsal lineage caused gastrulation defects, resulting in severe dorsal phenotypes. (M,P) Co-injection of *ca-rab7* mRNA rescued loss of function phenotype of morphant embryos. (Q) Quantification of results in K–P. (R,T) Tailbud control embryos, (S,U) siblings treated with *rab7* CRNP (S+L) or *rab7* SBMO showing dorsal phenotypes. (V,W) Quantification of results in (R,S and T,U). a, anterior; an, animal; ca, constitutive active; co, control; CRNP, Cas9 Ribonucleoprotein; d, dorsal; GD, gastrulation defect; p, posterior; SBMO, splice blocking Morpholino Oligonucleotide; st., stage; TBMO, translation blocking Morpholino Oligonucleotide; v, ventral; veg, vegetal; wt, wild type. Scale bars: 250 µm.

We speculated *rab7* could show distinct spatial enrichment of mRNA expression during early development. If the case, such enhanced abundance would give indications about tissue- and process-specific requirements. Indeed, expression analysis by *in situ* hybridization (ISH) revealed very dynamic spatial and temporal signals. Strong maternal expression was found in the animal half of cleavage stages, a signal detected until the onset of zygotic transcription after midblastula transition (MBT) (Fig. 1A,B; Fig. S1A). At early gastrula stages, transcripts were mainly found in the deep mesodermal ring, omitting the dorsal lip, i.e. the anterior/head part of Spemann-Mangold organizer (Fig. 1C,D; Fig. S1B). During late gastrulation, stronger signals were detected in the neural plate ectoderm and in the axial, notochordal mesoderm, latter of which continued to be positive for *rab7* during neurulation (Fig. 1E; Fig. S1C). By then, ectodermal expression became more restricted to the lateral neural plate, and later in the deep cells of the neural tube and brain tissues (Fig. 1F-H; Fig. S1D­-E). In following tailbud stages, *rab7* signals were detected in the cement gland and dermal areas, weaker in the notochord, and strong in the pronephric kidney, eyes, ventro-lateral neural tube, pharyngeal arches, trigeminal nerve complex, dorsal fin mesenchyme, and in the trunk neural crest cells (Fig. 1I,J; Fig. S1F,G; and data not shown). This analysis supported our hypothesis that *rab7* could be required for early embryonic development by participating in regulation of signaling activity in multiple tissues.

Next, we wanted to test an early *in vivo* requirement of *rab7* using a loss-of-function approach. We designed a morpholino oligomer (MO) targeting the 5′UTR of the L- and S-form of *Xenopus laevis rab7* to block translation of both homeologs (*rab7* TBMO). Morphant embryos passed through cleavage and blastula stages without detectable phenotypes (not shown). However, subsequent gastrulation movements were inhibited during gastrulation, causing complete failure to close the dorsal blastopore in about half of morphant embryos reaching early neurula stage (Fig. 1K,L). Other specimens displayed milder gastrulation phenotypes (not shown). The strong gastrulation defect became even more prevalent at tailbud stages, with further extension of the AP axis in control specimens, while morphant embryos remained wide open dorsally with strong dorsal curvature. By then, another 45% of specimens had developed milder phenotypes, recognizable mostly by impaired axial elongation (Fig. 1N,O,Q; and not shown). Importantly, co-injection of a constitutively-active (ca) *rab7* mRNA was able to rescue the strong gastrulation phenotype in a highly significant manner, demonstrating specificity of the observed MO effect. As tailbud stages, nearly all rescued embryos were able to close the blastopore and to elongate their AP axis, albeit with slightly reduced AP elongation (Fig. 1M,P,Q). To further underline specificity of this effect, we next designed single guide RNAs (sgRNA) to target the genomic loci of both *rab7* homeologs for CRISPR/Cas9-mediated mutagenesis, either in parallel, or individually. KO efficiency of injected embryos was determined by sequencing and subsequent analysis of indel distribution (Synthego ICE; for details see Materials and Methods). These analyses resulted in a predicted gene editing rate between 60% and 88% for L- or S-forms of the different sgRNAs (Fig. S2A-C). Genome editing of *rab7L*/S at the one-cell stage caused strong gastrulation defects, again resulting in tailbud stages with open dorsal part in 50% of specimens, resembling the phenotype shown for morphants (Fig. 1R,S,V). Interestingly, while selective targeting of homeolog L with sgRNA (L) also caused a similar phenotype in about 25% of injected specimen (Fig. S2D-F), no gastrulation defects were observed by only targeting homeolog S with sgRNA (S) (data not shown). Finally, we also designed a splice-blocking (SB) MO targeting the splice acceptor site at intron 2 of the *rab7* pre-mRNA to prevent splicing, and thus causing translational read-through and early termination (Fig. S2G). Successful inhibition of splicing could be demonstrated by RT-PCR for both homeologs, resulting in intron retention for each form (Fig. S2H). Injection of the *rab7* SBMO resulted in significant reduction of *rab7*-transcript amounts in morphant neurula or tailbud stage embryos, indicating nonsense-mediated decay of unspliced *rab7*, and thus successful knockdown of zygotic transcripts (Fig. S2I-L). Phenotypically, *rab7* SBMO injected embryos showed also gastrulation defects, but to a lower degree (Fig. 1T,U,W). Specimens that managed to close the blastopore were raised further. Such milder affected (or lower dose injected) morphants displayed deficits in AP elongation (Fig. S2O,P). Finally, by raising the surviving sgRNA (S+L), or low-dose *rab7* TBMO injected embryos, this late phenotype could be phenocopied in early tadpole stages, again supporting specificity of the effect (Fig. S2M,N,Q,R). In summary, our loss-of-function approach demonstrated a requirement of *rab7* for early embryonic development.

### Rab7 is required for axial mesoderm elongation and notochord morphogenesis

**Fig. 2.**
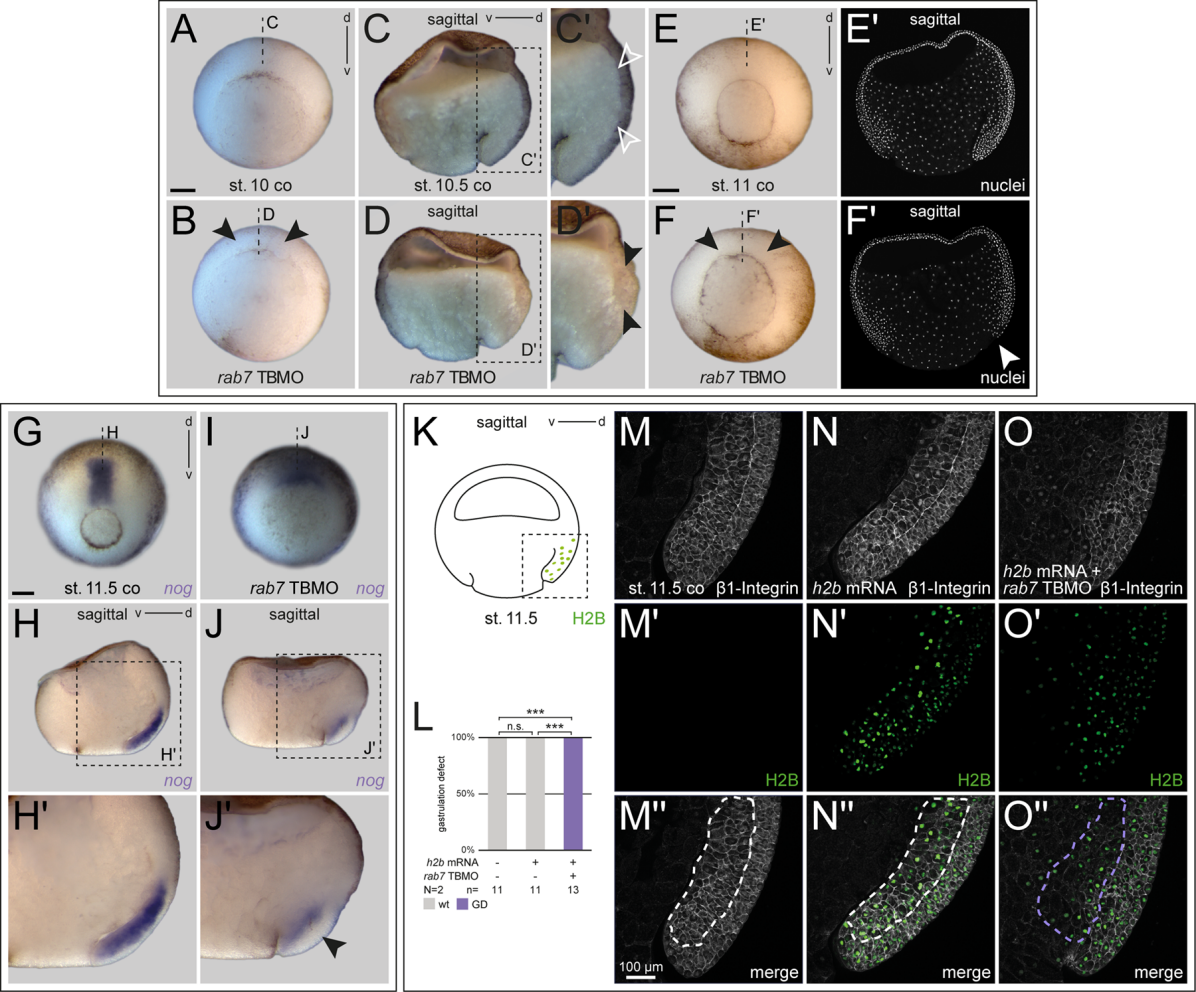
***rab7* morphants show impaired dorsal lip formation.** (A) Untreated embryos at the beginning of gastrulation forming a dorsal lip, (B) which can also be observed in *rab7* deficient siblings (black arrowheads). (C) In stage 10.5, sagittal sectioned control embryos revealed early involution movements. (C′) Enlargement of the area in the dashed black box in C, showing formation of Brachet's cleft was visible (white outlined arrowheads), (D) dorsal *rab7* knockdown specimen rarely showed involution in sagittal sections or Brachet's cleft formation, (D′) enlargement of the area in the dashed black box in D, (black arrowheads). (E) Older control embryo (mid-gastrula) with evenly shaped lip, (E′) sagittal section with Hoechst stained nuclei revealed proceeded dorsal involution and archenteron formation. (F) Same-age morphant siblings developed irregularly shaped lips around the blastopore (black arrowheads), and (F′) did not show any involuting tissue or archenteron formation in Hoechst stained sagittal section (white arrowhead). (G) Wild-type *nog* expression of control embryos and (I) reduced expression of *nog* and concomitant failed notochord elongation of dorsal *rab7* morphants. (H) Sagittal section and (H′, enlargement of the area in the dashed black box in H) exhibited proceeded notochord elongation marked by *nog* in untreated embryos. (J) Sagittal section and (J′) enlargement of the area in the dashed black box in J clearly depicted impaired *nog* expression (black arrowhead). (K) Schematic figure of sagittally bisected mid- to late gastrula embryo indicating view of dorsal lips shown in M-O″. (L) Quantification of overall embryonic phenotypes exemplified in M-O″. (M,N,O) bisected embryos stained for β1-Integrin (white). (M′,N′,O′) Histon 2B (H2B) GFP signal used as lineage tracer (green), (M″,N″,O″) merged channels. (M,N) Normal distributed β1-Integrin in wild-type dorsal lips or *h2b* mRNA-injected specimen, indicating target site. (O) Axial *rab7* morphant cells revealed altered shapes and lack of involution behavior and archenteron formation. Scale bars: A-J′, 250 µm; M-O″, 100 µm.

To better understand this gastrulation phenotype, we knocked down *rab7* specifically in the dorsal or ventro-lateral mesoderm. Thus for following experiments, *rab7* TBMO was co-injected along with a fluorescent lineage tracer specifically into the dorsal or ventral lineages, targeting the equatorial, i.e. mainly mesodermal progenitor areas (Fig. S3 for injection setup). Dorsal- or ventral-specific targeting was verified at early gastrula stages (Fig. S3A-J; see Materials and Methods). When mid-sagittal sections of dorsal *rab7* morphants were analyzed at early gastrula stages, such embryos formed a lip, but involution movements and archenteron formation were impaired (Fig. 2A-D; Fig. S3B-E). Interestingly, such bisections revealed a concomitant lack of Brachet's cleft, implying incorrect tissue remodeling during early gastrulation (Fig. 2C-F; Fagotto, 2020). These phenotypes became more apparent when morphants were analyzed for notochordal *noggin* (*nog*) expression at mid/late gastrula stage, illustrating strongly impaired axial elongation and reduced *nog* expression itself, also indicating a failure in maintaining notochordal fates in morphant tissue (Fig. 2G-J). Analysis of dorsal lips using an anti-β1-Integrin antibody further revealed altered cell shapes and impaired tissue rearrangement in the involuting marginal zone of morphants (deep layers), i.e. exactly in that mesodermal area where we found enrichment of *rab7*, paralleling the lack of axial elongation and archenteron formation (Fig. 2K-O).

**Fig. 3.**
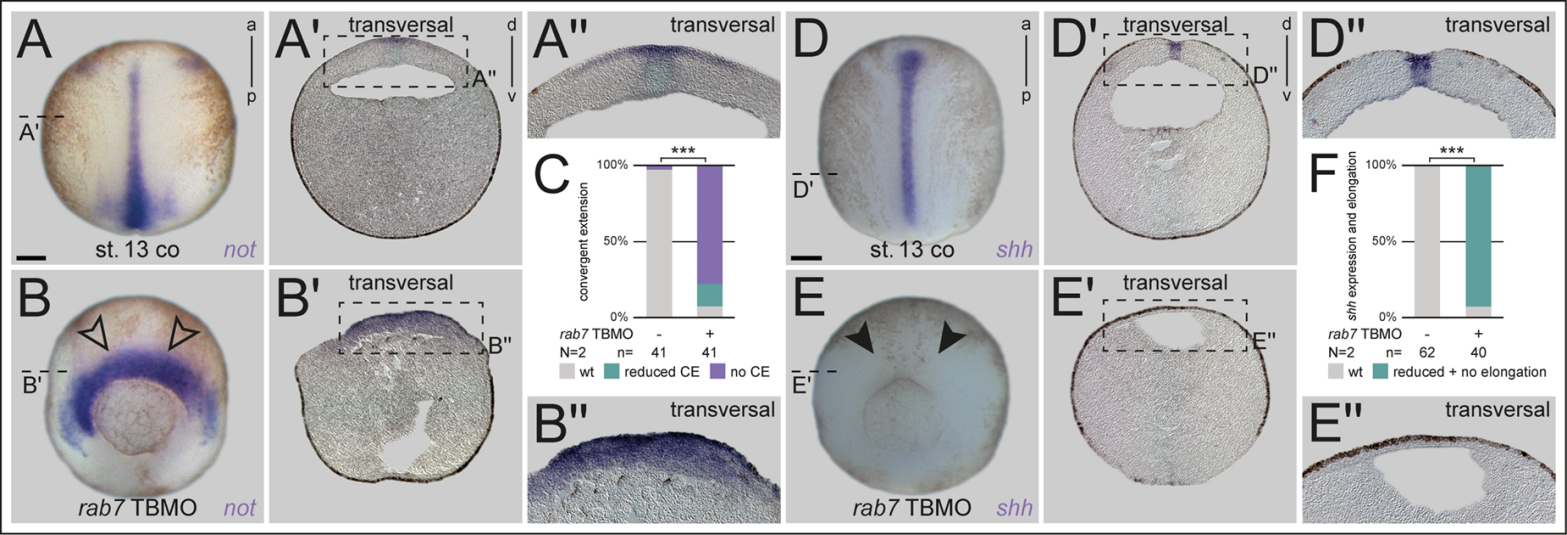
***rab7* is required for axial elongation and notochord morphogenesis.** (A,D) Early wild-type neurula embryos showing elongated notochords marked by the expression of *not* or *shh*. (A′,D′) Transversal sections indicated in (A,D) revealed *not* and *shh* expression in the axial neural plate and *shh* additionally throughout the whole notochord. (A″,D″) enlargement of the area in the dashed black boxes in A′ and D′, respectively. (B,E) Dorsal *rab7* TBMO injection resulted in embryos failing to elongate their notochord, although *not* expression intensity (black outlined arrowheads) was not reduced in comparison to *shh* (black arrowheads). (B′) Transversal section of morphant embryos showed lateral expanded expression of *not* above the dorsal lip and (E′) severely reduced *shh* expression. (B″,E″) enlargement of the area in the dashed black boxes in B′ and E′, respectively. (C,F) Quantification of results in A,B and D,E, respectively. Scale bars: 250 µm.

As these phenotypes indicated a failure in convergent extension (CE)-dependent processes, and thus, to form a proper elongated notochord subsequently, we examined notochord fate and appearance directly. When we checked expression of the marker *notochord homeobox* (*not*) in morphant embryos at early neurulation, lack of axial elongation was obvious, explaining the embryos’ inability to close the dorsal part of the blastopore (Fig. 3A-C). When using the *rab7* SBMO for dorsal knockdown, a milder but significant effect was observed as well (Fig. S4A-C). Interestingly, while *not* expression was not reduced in the axial mesoderm but extended into the lateral somitic areas, analysis of *sonic hedgehog* (*shh*) in the same experiments revealed a different effect. Morphant embryos showed similar inhibition of axial elongation but expression intensity of *shh* was reduced in most cases, again suggesting partial lack of notochordal fate (Fig. 3D-F).

Some milder affected morphant embryos were grown to tailbud stages to analyze notochordal tissue differentiation. In these stages, *not* expression was also not reduced, but appeared enhanced in the mid-trunk area of such specimens, where in wild-type embryos expression had already faded by that stage (Fig. S4D-E′). Overall, notochordal appearance in sagittal sections confirmed attenuated CE. Stronger affected specimens again developed open dorsal tissues, but mostly retained *not* expression, often split as two separated areas relocated medially (Fig. S4G). Staining embryos with an MZ-15 antibody, which detects outer keratan sulphates of the notochordal sheet, revealed absent or strongly reduced signals, supporting a lack of notochord differentiation (Fig. S4H-K). These phenotypic analyses showed that early *rab7* function is required for axial tissue morphogenesis and involution during gastrulation, and thus for subsequent notochord formation.

### Rab7 is required for dorsal mesoderm specification but not for organizer induction

**Fig. 4.**
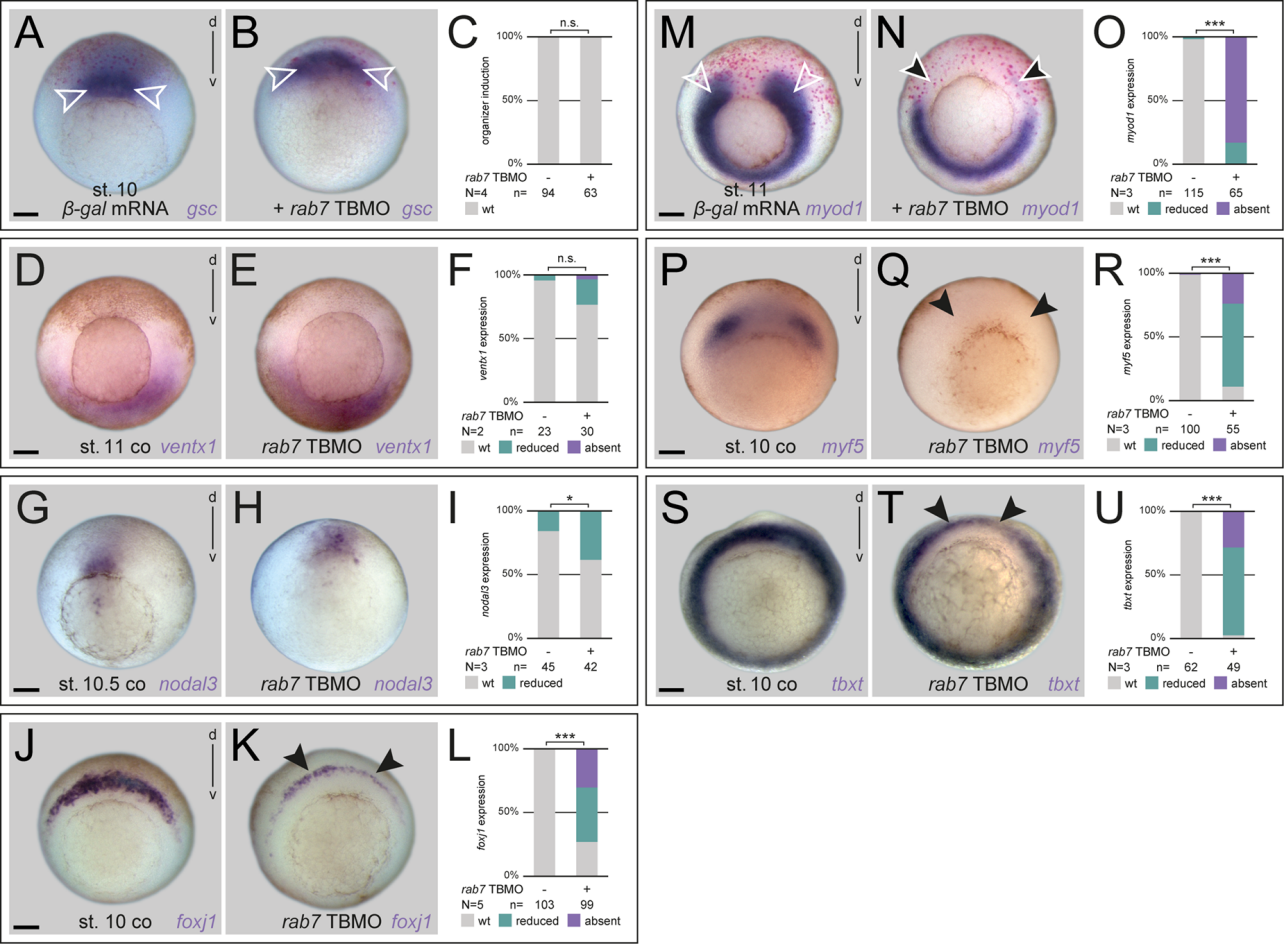
**Dorsal mesoderm specification requires Rab7 independent of the organizer.** (A,D,G,J,M,P,S) Untreated control specimen, or *β-gal* mRNA control injections confirming dorsal targeting (white outlined arrowheads in A,M) with wild-type *gsc*, *ventx1*, *nodal3*, *foxj1*, *myod1*, *myf5* and *tbxt* expression, respectively. (B,E) *rab7* loss of function in dorsal or ventral lineage did not alter *gsc* or *ventx1* expression, respectively. (H) *nodal3* expression was slightly reduced in some *rab7* morphant embryos. (K,N,Q,T) expression of *foxj1*, *myod1*, *myf5* and *tbxt* were severely affected in specimen with *rab7* deficiency (black arrowheads). (C,F,I,L,O,R,U) Quantification of results in A,B,D,E,G,H,J,K,M,N,P,Q,S,T. Scale bars: 250 µm.

To understand the role of *rab7* in this context, we next analyzed whether organizer induction was impaired by targeting this dorsal lineage. However, paralleling lack of *rab7* transcript enrichment in the anterior/head organizer area (Fig. 1C,D), neither knockdown with *rab7* TBMO or *rab7* SBMO, nor CRISPR-induced KO blocked organizer induction, as judged by *goosecoid* (*gsc*) and *chordin* (*chd*) expression (Fig. 4A-C; Fig. S5A-H). Next, we wanted to test if early, pre-gastrula dorso-ventral (DV) axis formation was altered in *rab7* morphants. Knockdown of *rab7* neither altered dorsal *chd*, nor ventral *ventx1* expression, indicating no alteration in Bone Morphogenetic Protein (BMP) gradient formation (Fig. S5I-K and Fig. S4D-F). This suggested that *rab7* is dispensable for both, endogenous organizer induction and DV patterning.

The reduced expression pattern of *shh* suggested that *rab7* might be selectively required for other dorsal mesoderm genes as well. Therefore, we next checked expression patterns of *nodal3* and *forkhead box J1* (*foxj1*), two Wnt-dependent marker genes expressed in the superficial mesoderm (SM), i.e. the outer layer of the trunk organizer tissue (Glinka et al., 1996; Smith et al., 1995; Stubbs et al., 2008; Walentek et al., 2013). Interestingly, while very early expressed *nodal3* was reduced only in a fraction of embryos, *foxj1* was strongly reduced after loss of *rab7* (Fig. 4G-L). Further, dorso-lateral markers *myogenic differentiation 1* (*myod1*) and *myogenic factor 5* (*myf5*) (Kjolby and Harland, 2017; Shi et al., 2002) were strongly downregulated in their paraxial expression domains at early and mid-gastrulation (Fig. 4M-R). Co-injected β-gal lineage tracer suggested this to be a cell-autonomous effect, as the ventro-lateral aspect of *myod1* (i.e. ventral lineage derived; compare Fig. S3I) was never inhibited in this injection approach (Fig. 4M-O). Together with the lateral extension of *not* into these areas, this indicated a potential shifting of paraxial fates (Fig. 3B). Finally, analysis of general mesoderm identity using *T-box transcription factor T* (*tbxt*, also known as *brachyury*) revealed a significant reduction of expression after *rab7* knockdown in this area as well (Fig. 3S-U), explaining the morphogenetic phenotype in the axial mesoderm (Figs 2 and 3). The selective down-regulation of some marker genes suggested a specific requirement of *rab7* for dorsal mesoderm development, probably downstream or in parallel of endogenous organizer induction.

### Rab7 is required for specification of the ventro-lateral mesoderm

**Fig. 5.**
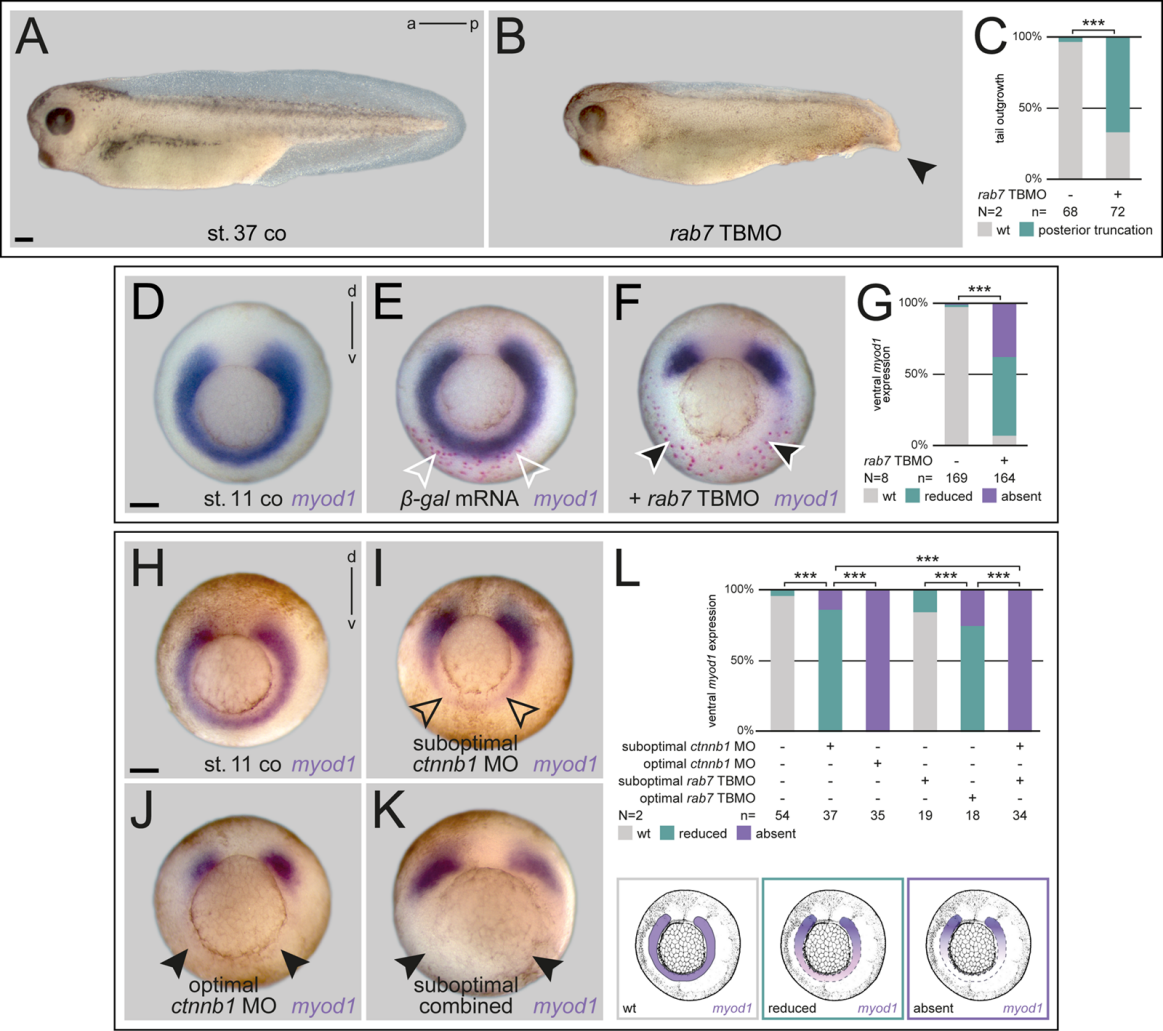
**Specification of the ventral mesoderm requires Rab7.** (A) Untreated early tadpoles with normal tail development, which was impaired by *rab7* loss of function in ventral lineage resulting in posterior truncations (B, black arrowhead). (C) Quantification of results in A,B. (D) Mid-gastrula control embryo depicting wild-type *myod1* expression, (E) injected *β-gal* mRNA marks ventrally targeted area (outlined white arrowheads). (F) Co-injection of *β-gal* mRNA and *rab7* TBMO revealed reduced or absent ventral *myod1* expression in most specimen at corresponding target site (white outlined black arrowheads). (G) Quantification of results in D-F. (H) Ventro-lateral mesoderm shown by wild-type *myod1* expression. (I) Ventral injection of suboptimal doses of *ctnnb1* MO resulted in minor reduction of *myod1* (black outlined arrowheads), (J) whereas optimal doses caused complete loss of ventro-lateral expression (black arrowheads). (K) Parallel injection of *rab7* TBMO and *ctnnb1* MO, both in suboptimal doses, lead to absence of *myod1* (black arrowheads). (L) Quantification of epistatic function of Rab7 and Ctnnb1 in ventro-lateral mesoderm, different manifestations of *myod1* expression exemplified below. Scale bars: 250 µm.

Organizer induction was not blocked after loss of *rab7*, yet, dorsal mesoderm specification was significantly impaired. Therefore, we asked if *rab7* also participated in subsequent ventro-lateral mesoderm specification, a process known to be dependent on zygotic *wnt8a* (Hoppler and Moon, 1998). During gastrulation, the organizer secretes Wnt antagonists dorsally in the axial mesoderm, while *wnt8a* activity is limited to the ventro-lateral mesoderm. Using targeted injections on the ventral side, we blocked *rab7* only in this area to test the possibility that it was required for mesoderm specification (Fig. S3F-J). Morphant embryos developed mild gastrulation phenotypes with low lethality rates, and thus the majority could be raised until tadpole stages. Such embryos showed ventro-posterior malformations, which became more pronounced as early tadpoles, when tail formation was strongly inhibited in most cases (Fig. 5A-C; Fig. S6A,B). Next, we analyzed ventro-lateral gene expression during late gastrulation, to test whether *rab7* was required for specification of these fates. Expression of *myod1* and *T-box 6* (*tbx6*), two of such marker genes, were strongly inhibited or lost in morphants, demonstrating a requirement of *rab7* for ventral mesoderm identity as well. Co-injection of β-gal lineage tracer demonstrated loss of expression only in the targeted part of the mesoderm, not dorso-laterally, i.e. again supporting a cell-autonomous effect on signal perception (Fig. 5D-G; Fig. S6C-E). To get a first indication, whether this lack of ventral specification is related to inhibition of endogenous Wnt signaling in the mesoderm, we performed epistasis experiments using suboptimal doses of the *rab7* TBMO in combination with a well-established *ctnnb1* (*β-catenin*) MO (Heasman et al., 2000). While injection of effective doses of *ctnnb1* MO resulted in loss of *myod1* expression, i.e. phenocopying, *rab7* morphants (Fig. 5J,L), low-dose injections of either *ctnnb1* MO or *rab7* TBMO both had only minor effects on ventral *myod1* (Fig. 5I,L). When both MOs were combined using low doses, *myod1* expression was lost in all double-morphants examined, supporting the conclusion of an epistatic interaction of Rab7 and Ctnnb1 (Fig. 5K,L). Together, our experiments support the conclusion that *rab7* participates in specification of ventral mesodermal fates during gastrulation, potentially by regulating endogenous Wnt8a-activated signaling.

### Rab7 acts epistatically with the endosomal regulator Vps4

**Fig. 6.**
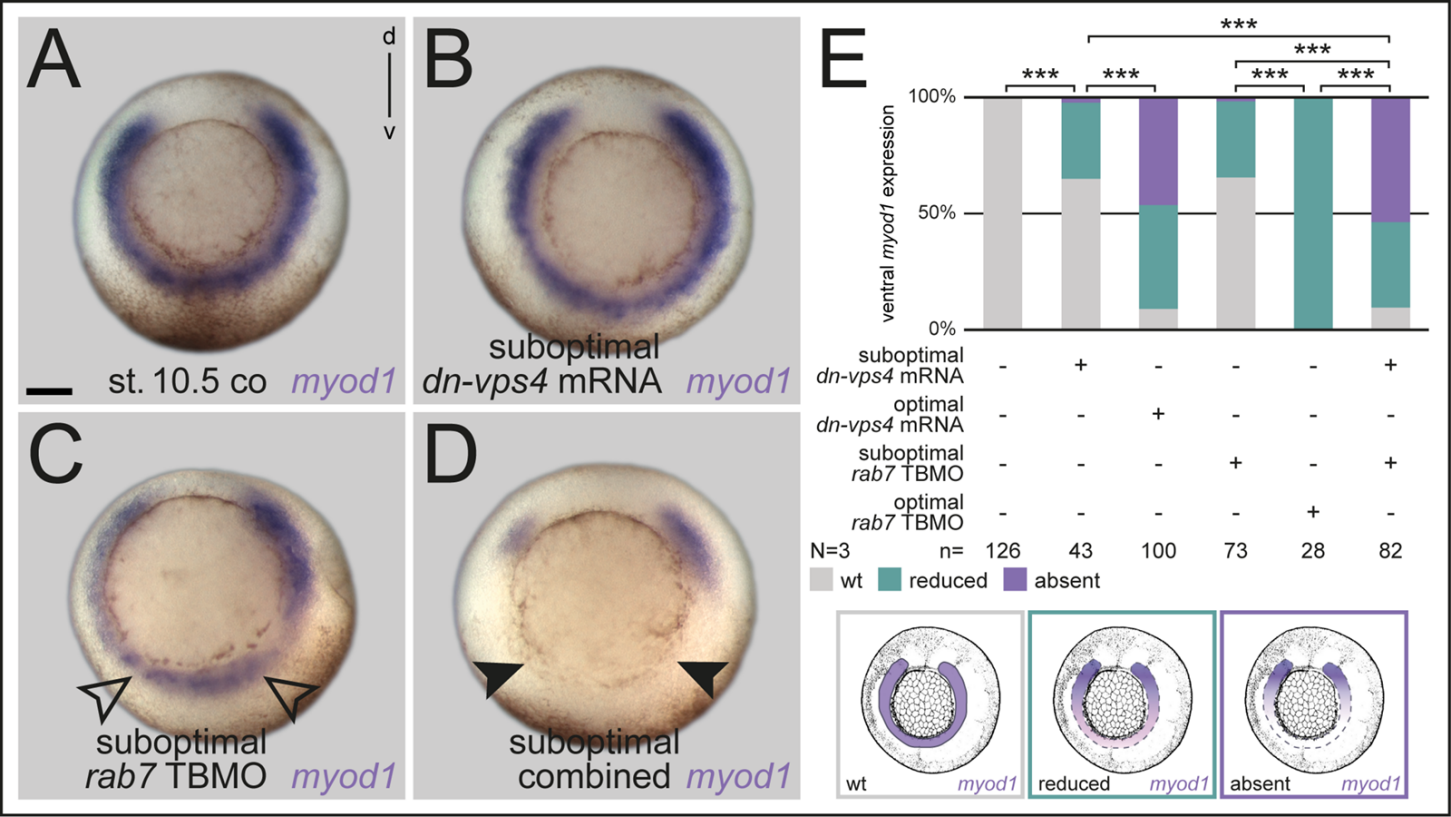
**Small GTPase Rab7 acts epistatically with the LE regulator Vps4.** (A) Untreated specimen; (B) Injection of *dn-vps4* mRNA or (C) *rab7* TBMO in suboptimal dose led to minor reduction of *myod1* expression on ventral side (black outlined arrowheads in C). (D) Parallel suboptimal injection of *rab7* TBMO and *dnvps4* mRNA resulted in absent *myod1* expression (black arrowheads). (E) Quantification of results, different manifestations of *myod1* expression in analysis are exemplified below quantification. Scale bar: 250 µm.

In most contexts, Rab7 acts via its well-studied role as a regulator of late endosomal function. However, in some cases it has been shown to perform a cellular role independent of LE, and, potentially not in the endo-lysosomal pathway (Guerra and Bucci, 2016). Therefore, we aimed to address this point as well, by testing if other late endosomal regulators, which have also been demonstrated to be required for Wnt function, regulate ventral fates in concert with Rab7. We chose two components of the ESCRT machinery (Horner et al., 2018), which have previously been characterized in Wnt signal transduction in *Xenopus*, i.e. *hepatocyte growth factor-regulated tyrosine kinase substrate* (*hrs*) and *vacuolar protein sorting 4 homolog* (*vps4*) (Taelman et al., 2010). Using doses that have been shown to block double axis formation, we knocked down *hrs* ventrally*,* targeting the ventral mesoderm (Fig. S6F-H), or overexpressed a dominant-negative version of *vps4* (*dn-vps4*) (Bishop and Woodman, 2000), to analyze LE-dependent mesoderm patterning. In both cases, loss of these regulators caused strong reduction of *myod1* in the ventral part, supporting a necessity for correct LE, and possibly Wnt function in this tissue (Fig. 6A,E; Fig. S6F-H). As this implicated a functional cooperation with Rab7 on LE, we performed an epistatic analysis to demonstrate interaction. Either low-dose injection of *dn-vps4* mRNA, or that of *rab7* TBMO caused minor reduction of *myod1* expression, however, parallel injection of both caused strong inhibition of *myod1* in a significant manner (Fig. 6B-E). From these results we conclude that Rab7 should act in a canonical manner as an endosomal regulator in the ventral mesoderm, together with LE effectors known to be required for Wnt activation.

### Rab7 is required for canonical Wnt pathway activation *in vivo*

**Fig. 7.**
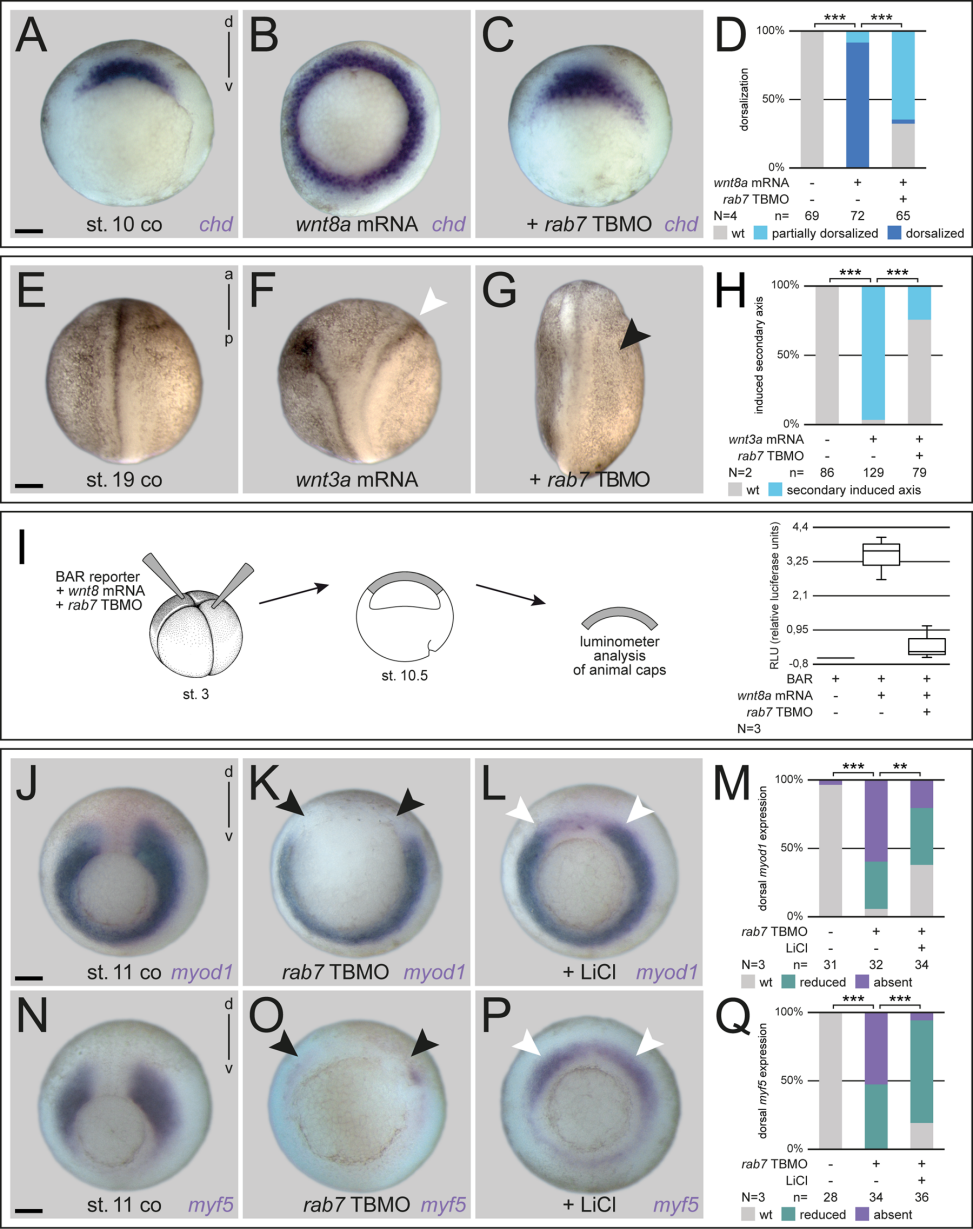
**Rab7 is required for canonical Wnt pathway activation.** (A) Wild-type dorsal expression of *chd* in comparison to specimen with (B) radial injected *wnt8a* mRNA showing an extended *chd* expression domain around the blastopore. (C) Co-injection of *rab7* TBMO restricted additional *chd* expression to normal wild-type state. (D) Quantification of results in A-C. (E) Untreated control specimen, (F) in comparison to induced double axis (white arrowhead) after ventral *wnt3a* mRNA injection, (G) parallel injection of *rab7* TBMO inhibited secondary axis formation (black arrowhead). (H) Quantification of results in E-G. (I) Luciferase-based Wnt reporter assay analysis at stage 10.5 illustrated that *wnt8a* induced reporter activity was blocked by *rab7* loss of function in animal caps. (J,N) Wild-type expression of *myod1* and *myf5* in stage 11 control embryos. (K,O) Absent dorsal *myod1* expression and lost domains upon *rab7* loss of function (black arrowheads). (L,P) Treatment with LiCl partially rescues dorsal expression of *myod1* and *myf5* in *rab7* morphant embryos (white arrowheads). Please note that LiCl-induced ectopic expression in the axial mesoderm was not inhibited by the *rab7* TBMO. (M,Q) Quantification of results in J-L and N-P, respectively. Scale bars: 250 µm.

In order to investigate directly whether the loss of *rab7* interfered with endogenous Wnt signals in the mesoderm, and to bypass the possibility that any putative maternal *rab7* mRNA or protein would ‘cover’ its requirement for organizer induction in our experiments, we activated Wnt signaling exogenously. First, we used radial injections of *wnt8a* mRNA, which is well known to dorsalize the embryo (Hikasa and Sokol, 2013; Smith and Harland, 1991). Injections caused radial expression of dorsal-specific organizer genes and erased that of ventral-specific *ventx1* (Fig. 7A,B,D; Fig. S7A-H). Importantly, co-injection of *rab7* TBMO restored the DV-axis highly significantly, again without impacting on endogenous organizer-specific expression of *chd* or *nog* (Fig. 7C,D; Fig. S7C,D). Early gastrula embryos tested for *ventx1* showed phenotypic restoration of their DV axis, yet *ventx1* expression stayed partially reduced (Fig. S7G,H). These results suggest that *rab7* is required for ligand-dependent activation of Wnt signaling, upstream of exogenously induced organizer genes.

If *rab7* participated in Wnt pathway activation *in vivo*, then knockdown should prevent induction of secondary axes, a classical readout for canonical Wnt pathway in *Xenopus* (Sokol et al., 1991). Co-injection of *rab7* TBMO was sufficient to prevent *wnt3a*-induced double axis induction in a highly significant manner (Fig. 7E-H). Importantly, the efficiency of *ctnnb1* to induce double axes was not altered after *rab7* knockdown (Fig. S7J-M). To further rule out the hypothesis that this effect was due to impairment of processes downstream of organizer induction, we analyzed these secondary organizers for expression of *gsc*, which clearly demonstrated inhibition of Wnt-induced organizers (Fig. S7N-P). These results were further supported using the Wnt-specific β-catenin activated reporter (BAR) (Biechele et al., 2009) in animal caps, where *wnt8a*-induced activation was also blocked by *rab7* inhibition (Fig. 7I). The same reporter blockage after loss of *rab7* was found endogenously, when the BAR construct was directly injected into the Wnt-dependent ventral mesoderm. Loss of *rab7* reduced signals by 90%, indicating that the loss of ventral markers was caused by Wnt pathway inhibition upstream of *β-catenin*-mediated transcriptional activation (Fig. S7I). In a final approach, we wanted to test if induced activation of zygotic Wnt signaling was sufficient to rescue the loss of paraxial marker genes after *rab7* knockdown. Thus, we incubated dorsal morphants in 0.2 M LiCl at the begin of gastrulation, i.e. after organizer induction. For both, *myod1* and *myf5*, LiCl treatment was partially able to rescue expression in the paraxial mesoderm, while LiCl-induced ectopic expression in the axial areas was not inhibited by inhibition of *rab7*, supporting a role upstream of β-catenin activated transcription (Fig. 7J-Q). These results support the conclusion that *rab7* participates in mediating early *Xenopus* Wnt signals in a context-dependent manner. Altogether, we therefore conclude from our experiments that loss of the small GTPase Rab7 can interfere with Wnt pathway activation in early frog embryos, upstream of Ctnnb1 stabilization.

## DISCUSSION

In this study, we analyzed the *in vivo* role of the small GTPase Rab7 in the frog *Xenopus* with focus on its role in early patterning and regulation of morphogenetic processes during gastrulation. We were able to demonstrate a requirement for dorsal and ventral gastrulation processes, which were both strongly impaired after loss of *rab7*. Further, our results implicate that Rab7 participates in mesodermal patterning processes in the early embryo, at least partially in a Wnt-dependent manner, explaining the observed morphogenetic phenotypes.

### *rab7* has distinct activity pattern throughout embryonic development

In developmental studies, genes with categorized housekeeping function are mostly used as molecular tools – developmental expression is rarely included, as it is considered to be ubiquitous, with little temporal or spatial fluctuations by definition (‘housekeeping genes’, Hounkpe 2021; Kim et al., 2012; Lee and Harland, 2010 for Rab coding examples). Our analysis of *rab7* revealed strikingly dynamic and spatially restricted expression patterns throughout early development, suggesting a tissue- and context-specific requirement. Therefore, we would predict that many other of such classified genes show tissue-specific and developmentally regulated function as well, and should therefore be used carefully and classified as strict housekeeping genes. Alternatively, for clarity, such examples could be given a separate subcategory within the term ‘housekeeping gene’. In context of membrane trafficking, expression of different endosomal regulators (e.g. *hrs* or *vps4*) could reveal distinct *endosomal synexpression groups*, following a concept proposed before (Niehrs and Pollet, 1999). Such analyses could reveal novel roles for endosomal regulation of developmental processes or pathways, in line with a shared co-transcriptional regulation, as demonstrated for genes coding endosomal components (Ploper et al., 2015; Sardiello et al., 2009). In the case of *rab7*, strong signals in the neural plate border (Fig. 1F-H), pronephric kidney, and cranial and trunk neural crest (Fig. 1I,J) suggest such possible roles, also in other Wnt-regulated tissues (Borday et al., 2018; Burstyn-Cohen et al., 2004; Honoré et al., 2003; Pla and Monsoro-Burq, 2018; Villanueva et al., 2002; Zhang et al., 2011).

### Rab7 is required for dorsal mesoderm specification and tissue remodeling during gastrulation

We performed loss of function of *rab7* using antisense oligos and CRISPR/Cas9-mediated genome editing. Reflecting the late expression in diverse tissues, mildly affected, or low-dose injected tadpoles displayed shortened AP axes, head and eye defects, and edema formation, the latter putatively due to loss of *rab7* in the pronephric system (Fig. 1; Fig. S2; Wessely and Tran, 2011). Yet, while developing no phenotypes during cleavage or blastula stages, the most prominent developmental defects of *rab7*-deficient embryos became apparent during gastrulation, as a result of incorrect mesoderm patterning. The lack of very early phenotypes, despite the presence of a large supply of maternal *rab7* mRNA in cleavage and blastula stages suggest that this pool might also be required after MBT, i.e. at least partially during gastrulation. This is supported by the fact that the *rab7* TBMO was more efficient in causing gastrulation defects than the *rab7* SBMO.

Interestingly, work in mice supports evolutionary conservation of the role of *rab7* in this context (Kawamura et al., 2012). Here, *rab7* KO also prevented gastrulation, resulting in early embryonic death. More importantly, the authors demonstrated recently that this phenotype was caused by lack of proper mesoderm formation, recognizable by defects in tissue remodeling and subsequent germ layer formation (Kawamura et al., 2020). These cellular phenotypes are highly reminiscent of our observations in the dorsal mesoderm after loss of *rab7*, where cellular arrangements were disorganized as well, and correct formation of Brachet's cleft was impaired, i.e. germ layers also failed to separate correctly (Fig. 2; Fig. S4). Another indication for a conserved role of *rab7* in these processes might be the alteration of cell adhesion we observed (Fig. 2K-O). In the mouse, such altered cell adhesion was also observed after loss of *rab7*, paralleling the failed tissue remodeling (Kawamura et al., 2020).

From work in cell culture it is known that Rab7 is required for correct activation and localization of β1-Integrin at the cell membrane in a permissive way, i.e. as a component required for transport towards the membrane (Arjonen et al., 2012; Margiotta et al., 2017). Thus, such a function of Rab7-mediated transport could specifically explain changes of β1-Integrin after *rab7* inhibition (Fig. 2O). Yet, we cannot clearly distinguish whether the alterations of cell adhesion in the dorsal mesoderm is a direct result of loss of Rab7-mediated transport of adhesion components, or an indirect consequence of the lack of mesodermal specification. The significant downregulation of *tbxt* in most embryos argues for the second possibility (Fig. 4S-U), which suffices to explain the observed problems of tissue remodeling, axial elongation and notochord morphogenesis, since Tbxt is a well-known upstream regulator of mesoderm specification and non-canonical Wnt pathways required for CE (Bruce and Winklbauer, 2020; Schulte-Merker and Smith, 1995; Tada and Smith, 2000).

### Rab7 mediates dorsal development independent of the organizer

Strikingly, we did not observe a change in organizer induction as judged by expression of *gsc* or *chd* at early gastrulation. Whether such a Rab7-deficient organizer is fully functional, i.e. capable of inducing a secondary axis in a classical transplantation assay, is not clear from these analyses. Yet, embryos show no signs of ventralization or dorsalization, neither phenotypically, nor when analyzed for mid-gastrula expression of DV-specific genes *ventx1* or *chd* (Fig. 4D-F; Fig. S5F-K). These results argue against an involvement of *rab7* in early Wnt-mediated Nieuwkoop center/organizer induction (Fagotto et al., 1997; Heasman et al., 1994) or in transforming growth factor beta (TGF-β)/Nodal pathway-induced activation of organizer genes, nor in BMP-mediated ventral development (De Robertis, 2009). These conclusions are supported by the recent report also showing no alteration of Nodal or BMP signaling in *rab7* KO mice (Kawamura et al., 2020). So far, neither involvement of Rab7, nor of LE in general has been linked to activation of TGF-β signaling, a finding we would also conclude from our *Xenopus* analyses.

In contrast to wild-type expression of organizer genes, we found that *rab7* was clearly necessary for *foxj1, myod1, myf5,* and *tbxt*, and partially for correct *nodal3* expression (Fig. 4G-U). Most of these genes are known to depend on active Wnt signaling (with uncertainty for *tbxt*), however, to what extent maternally or zygotically activated Wnt signals contribute to their activation is not fully understood (Shi et al., 2002; Smith et al., 1995; Stubbs et al., 2008; Vonica and Gumbiner, 2002; Walentek et al., 2013). *nodal3* expression, which was impacted least after *rab7* inhibition, is initiated just after MBT and thought to be a direct Wnt target (Glinka et al., 1996; Smith et al., 1995). Therefore, these results might indicate a role for Rab7 only for processes relying on zygotic Wnt activation. In the recently analyzed *rab7* KO mouse, gastrulation phenotypes and lack of *tbxt* expression have been demonstrated as well, and these phenotypes were related to inhibition of Wnt signaling by reduced degradation of the Wnt antagonist Dickkopf (Dkk) (Kawamura et al., 2020). It is not clear whether Rab7 participates in Dkk degradation in *Xenopus* development. However, excess Dkk protein caused by loss of Rab7 can neither explain inhibition of induced double axes, nor blockage of transcriptional Wnt reporter activation in animal caps, which are devoid of *dkk* transcripts (Fig. 7; Glinka et al., 1998). Further, *dkk* is not expressed endogenously in the ventral mesoderm, i.e. accumulated protein can therefore not explain *rab7* knockdown-induced loss of ventral marker genes (Fig. 5; Fig. S6). We therefore conclude that at least in these contexts, Rab7 should participate in Wnt ligand-induced signal transduction, probably as part of a late endosomal machinery-mediated signal maintenance mechanism (Fig. 6, Taelman et al., 2010, and below).

### Rab7 is necessary for Wnt activation in *Xenopus* in a context-dependent manner

The unexpected lack of ventralization phenotype contradicted the reported role of LE for Wnt pathway activation, as we expected Rab7 to be also required for maternal, Wnt-dependent organizer induction (Taelman et al., 2010; Vinyoles et al., 2014). This was even more puzzling, as we could demonstrate an absolute requirement for exogenously induced activation of Wnt-dependent processes, i.e. double axis assay, Wnt reporter activation, and the restoration of the DV axis after Wnt8a-mediated dorsalization (Fig. 7; Fig. S7). The last result exemplified this discrepancy, as exogenously induced Wnt-dependent dorsal fates were blocked after loss of *rab7*, while the endogenous, organizer-induced expression of *chd* and *nog* stayed unaltered (Fig. 7A-D; Fig. S7A-D). A compensatory action by the paralogous *rab7b* can be excluded, as it is not present in the early embryo (Peshkin et al., 2019 preprint; Session et al., 2016; our preliminary results). One straightforward explanation would be that endogenous maternal Wnt activation does not require ligand-mediated receptor activation and endocytosis, which we used for exogenous Wnt pathway induction. This could include a mechanism bypassing the endolysosomal system, what would be in agreement with the fact that injection of *dickkopf-1* mRNA, which blocks extracellular Wnt receptor activation, does not ventralize the embryo either (Glinka et al., 1998). In cell culture and *Xenopus* embryos, LE have been reported to be necessary to establish a robust Wnt output, i.e. to maintain continuous inhibition of glycogen synthase kinase 3 (GSK3), and thus Wnt pathway activation. However, endogenous organizer induction has not been analyzed in these experiments (Niehrs and Acebron, 2010; Taelman et al., 2010). In fact, we cannot exclude that early Wnt activation might only rely on ‘fast-acting’, LE-independent mechanisms (like Axin inhibition), which have been suggested to be required for GSK3-inhibition and Wnt activation (Clevers and Nusse, 2012; Li et al., 2012). Alternatively, the *Xenopus* zygote might already contain fully or partially matured LE, as proposed before, whose functionality we are not able to interfere with using embryonic injections (Dobrowolski and De Robertis, 2011).

In contrast to the rather complex involvement of Rab7 for dorsal fates, we could show a requirement for endogenous ventro-lateral mesoderm patterning, as all marker genes (*myf5, myod1, tbx6*) were strongly downregulated after loss of *rab7* (Fig. 4M-R, Fig. 5D-G; Fig. S6C-E). These genes have all been shown to depend on a ventral source of Wnt, mediated by Wnt8a (Hoppler and Moon, 1998; Hoppler et al., 1996; Kjolby and Harland, 2017; Shi et al., 2002), which was supported by the epistatic effect of *rab7* knockdown with *ctnnb1* knockdown (Fig. 5H-L). Furthermore, we could also demonstrate that *rab7* knockdown blocked endogenous Wnt target gene activation, as monitored using a ventral mesodermal BAR reporter signal (Fig. S7I). From these experiments, we suggest that *rab7* participates in ventral mesoderm specification, at least partially as a necessary factor for Wnt target gene activation. This effect on mesoderm specification was phenocopied by loss of LE-associated ESCRT factors *vps4* and *hrs*, which are also known to be required for Wnt pathway activation (Taelman et al., 2010). As we could demonstrate an additive relationship with loss of *rab7* (Fig. 6), we suggest Rab7 fulfills this role as an endosomal regulator required for correct LE-mediated Wnt transduction (Dobrowolski and De Robertis, 2011; Hikasa and Sokol, 2013).

### The connection of Rab7 to other signaling pathways – beyond Wnt activation

Our findings implicate a role of Rab7 for exogenously induced Wnt activation, and for endogenous ventral mesoderm specification, probably in a Wnt-dependent function. Yet, some results obtained with our dorsal analyses might indicate further, potentially Wnt-independent roles during gastrulation. While dorsal loss of *rab7* resulted in subsequent gastrulation and axial elongation defects, selective downregulation of *shh* seems puzzling (Fig. 3). Regulation of *shh* in the ventral neural tube is well analyzed (Dessaud et al., 2008), however, little was reported about the induction and maintenance of its notochordal expression. Activin was shown to be able to induce *shh* in animal caps, but not endogenously in the mesoderm (Yokota et al., 1995), and we have no evidence for a participation of Rab7 in TGF-β pathways either. Yet, in the well-studied limb bud, Wnt7a has been shown to be required for induction and/or maintenance of *shh*, offering a potential link to our observations (Parr and McMahon, 1995; Yang and Niswander, 1995). Another interesting aspect also comes from the limb bud, where fibroblast growth factor (FGF) signaling was reported both to induce and to maintain *shh* expression (Scherz et al., 2004; Vogel et al., 1996; Yang and Niswander, 1995). FGF signaling is known to cooperate with Wnt in different contexts, and both are required additively to induce *myf5* in the somitic mesoderm (e.g. Shi et al., 2002). Thus, if *rab7* additionally participated in FGF pathway activation, this would explain the strong effect on *myf5* and *myod1* after loss of function (Fig. 4M-R), the partial ability to rescue *myf5* and *myod1* by LiCl treatment (Fig. 7J-Q), and the differential impact on *nodal3* versus *foxj1* and *tbxt* (Fig. 4G-L and S-U). The last three genes depend on Wnt signaling, but only *tbxt* and *foxj1* have also been shown to be regulated by the FGF pathway dorsally, downstream of Nodal3-induced activation of Fgf receptor 1 (Glinka et al., 1996; Schneider et al., 2019; Smith et al., 1995; Vick et al., 2018; Yokota et al., 2003).

## MATERIALS AND METHODS

### *Xenopus laevis* care and maintenance

Frogs were purchased from Nasco (901 Janesville Avenue P.O. Box 901 Fort Atkinson, WI, USA). Handling, care and experimental manipulations of animals was approved by the Regional Government of Stuttgart, Germany (V349/18ZO ‘Xenopus Embryonen in der Forschung’), according to German regulations and laws (§6, article 1, sentence 2, number 4 of the animal protection act). Animals were kept at the appropriate conditions (pH=7.7, 20°C) at a 12 h light cycle in the animal facility of the Institute of Zoology of the University of Hohenheim. Female frogs (4-12 years old) were injected subcutaneously with 300-700 units of human chorionic gonadotropin (hCG; Sigma-Aldrich), depending on weight and age, to induce ovulation. Sperm of male frogs was gained by dissection of the testes that was stored at 4°C in 1× MBSH (Modified Barth's saline with HEPES) solution. Embryos were staged according to Nieuwkoop and Faber (1994). Only clutches of embryos from healthy females were used for the experiments reported here, provided the early embryonic stages showed normal survival rates as well. Individual embryos from one batch were randomly picked and used either as control or test specimens. If control groups displayed unusual developmental defects later in development, such clutches were excluded as well, based on empirical judgement.

### Lineage-specific microinjections

For lineage-specific experiments, embryos were injected at the four-cell stage into the marginal (equatorial) region of either both dorsal or both ventral blastomeres, to target the dorsal or ventro-lateral mesoderm specifically (Fig. S3A,F). Using a Harvard Apparatus setup, drop size was calibrated to 4 nl per injection. For verification of dorsal-specific or ventral knockdown, a lineage tracer (fluorescein-dextran, mGFP mRNA, H2B-GFP mRNA, or β-gal mRNA) was added. For both targeted injections into the dorsal and ventral mesoderm, embryos were cultivated until early gastrula stages (stage 10-10.5), when the DV axis is easily recognized by dorsal lip formation, and verified for correct targeting of dorsal or ventral lineages, respectively (Fig. S3A-J). In dorsal experiments analyzing somitic (paraxial) marker genes (*myod1*, *myf5*), injections were targeted slightly more lateral into the same blastomere (not shown). For experiments with analyses at mid to late gastrula (stage 11-12,5), embryos were checked at early gastrula, then fixed at later stages (Fig. S3D-E″,I-J″). For all targeted knockdown analyses, embryos of all treatments (uninjected specimens of the same batch, and control-injected or treatment-injected specimens) were cultivated for exactly the same time under the same temperature-controlled conditions before fixation and analysis. Thus, any apparent differences in staging within one experiment should represent phenotypic alterations caused by the treatment itself.

### Morpholino design and microinjections

The *rab7a*-5′UTR-/TBMO was designed using the sequence of the S-form from the genomic sequence as deposited in gene bank (one mismatched base pair for the L-Form; no binding to *X. laevis rab7b* mRNA). TBMO-sequence is 5′-GTCTCCGCTTCCTACCCCTGCCAGC-3′. The *rab7a* SBMO was designed using the sequence of the L-Form from the genomic sequence as deposited in gene bank (three mismatched base pairs for the S-Form). Splice acceptor site at intron 2 of the *rab7a* pre-mRNA is targeted by SBMO (5′-GCCAACCCTAGAATGGAAGATACAA-3′). Further MOs used in this study were *ctnnb1* and *hrs* MO as published (Heasman et al., 2000; Taelman et al., 2010) or a random co-MO as a MO fill up for epistatic analyses. Total amounts of injected MOs were: 0.4 pmol *ctnnb1* MO (suboptimal dose), 0.8 pmol *ctnnb1* MO (optimal dose), 1.6-2.0 pmol *hrs* MO, 0.7 pmol *rab7* TBMO (suboptimal/low dose), 1.0-1.4 pmol *rab7* TBMO (optimal dose), 1.4-4.0 pmol *rab7* SBMO.

### mRNA synthesis and microinjections

Plasmids were linearized with NotI and transcribed *in vitro* (Sp6 polymerase) using Ambion message machine kit. Drop size was calibrated to 4 nl per injection. Total amounts of injected mRNA per embryo are as followed: 80 pg *ca-rab7a* (*Canis lupus*) mRNA, 400 pg *dn-vps4* mRNA, 400 pg mGFP mRNA, 400 pg H2B-GFP mRNA, 160 pg *wnt8a* mRNA, 400 pg β-gal mRNA.

### sgRNA design and microinjections

Two single-guide RNAs were designed for the *Xenopus laevis rab7a* gene, *rab7* CRNP (S+L), target sequence: 5′-GGTGATGGTGGATGACAGATTGG-3′ (on exon 3), and rab7 CRNP (L), target sequence: 5′-GGGACACAGCTGGGCAGGAA AGG-3′ (on exon 4), using the publicly available ‘CRISPscan’ software. sgRNAs were transcribed with the MEGAshortscript T7 Kit from synthetic DNA oligomers and purified with the MEGAclear Transcription Clean-Up Kit (both ThermoFisher).

Oligos for synthesis were: rab7-CRNP (S+L) forward: 5′-GCAGCTAATACGACTCACTATAGGTGATGGTGGATGACAGATGTTTTAGAGCTAGAAATAGCAAG -3, rab7 CRNP (L) forward 5′-GCAGCTAATACGACTCACTATAGGGACACAGCTGGGCAGGAAGTTTTAGAGCTAGAAATAGCAAG-3′, and general reverse_5′-AAAAGCACCGACTCGGTGCCACTTTTTCAAGTTGATAACGGACTAGCCTTATTTTAACTTGCTATTTCTAGCTCTAAAAC -3′.

Embryos were injected with 1 ng Cas9 protein (PNA Bio) and 300 pg sgRNA at the one-cell stage and cultivated at room temperature (RT) until desired stage. DNA was isolated from ten mutant and five control embryos. For verification of successful genome editing, isolated DNA was proceeded by RT-PCR and sequenced. Analysis of sequenced DNA was analyzed via Synthego ICE. PCR-Primers for sequencing were the following: *rab7* CRNP(S+L), L-form (FP 5′-AGCCGTATTTCTTTGTTGTGCCA-3′; RP 5'-ATTCCAGGTGCAGTGAGATGT-3′), *rab7* CRNP(S+L), S-form (FP 5'-TGAGTGCATTGTGCTGTGTG-3′; RP 5′-CCCCCATTTGAAAACTGAAGAGAG-3′), *rab7* CRNP(L), (FP 5′ ACGGGAGCAGATTTAATAGGACA-3′; RP 5′-CTTGGACTCGCCTGGATGAG-3′).

### PCR-based verification of efficient intron retention for the SBMO

For verification of SBMO, a standard RT-PCR was performed. SBMO was injected in all four blastomeres of four-cell embryos, cultivated until stage 13, and fixed for RNA isolation. The following PCR primers were used: forward 5′-CCTCCAGGAATATGCAGGAA-3′; reverse 5′-CTGCATTGTGACCAATCTGTC-3′.

### Luciferase-based Wnt assay

For Luciferase-based Wnt assay (Promega Dual-Luciferase^®^ Reporter Assay System), embryos were injected into two animal or ventral blastomeres at the four-cell stage (80 pg BAR reporter DNA plus 40 pg Renilla DNA). For exogenously induced reporter activity, embryos were injected of 240 pg *wnt8a* mRNA +/− 1.4 pmol *rab7* TBMO and cultivated until stage 10.5, then animal caps were dissected and further processed. For endogenous analysis, embryos were co-injected with rab7 TBMO, cultivated until stage 12, then the ventral halves were dissected. Dissection of tissues was performed in 0.1× MBSH. Extracted tissues (ten animal caps or ventral halves) were transferred into lysis buffer (Promega) and incubated for 15 min. Lysates were centrifuged repeatedly for 15 min, then supernatant transferred in triplets onto a 96-well plate for Luciferase analysis by the GloMax Explorer system. Relative Luciferase units (RLU in %) were calculated by the ratio of Luciferase and Renilla values.

### *In situ* hybridization

For *in situ* mRNA detection, ISH was performed after fixation in MEMFA for 2-3 h at RT and processed following a customized standard protocol (Sive et al., 2000; customization after R. Rupp, personal communication). PCR primer pair for cloning of *X. laevis rab7a* were: forward: 5′-ATCAATACGCGTCAACAACC-3′; reverse: 5′ ACAGGTGTCAGTATTCATTTGG-3′. Cloned full length coding region was sequenced. *X. laevis rab7a* shows no significant overlap with *rab7b*, excluding mixed signals during ISH. RNA *in situ* probes were transcribed using SP6 or T7 polymerases.

### Axis induction assay

Double axis assay was performed by single injection of 0,8 pg *wnt3a* mRNA +/− 0,7 pmol *rab7* TBMO into one ventral mesodermal blastomere at the four-cell stage as described (Beyer et al., 2012). Embryos were raised until gastrulation or neurula stage. Double axes were scored empirically by second ventral *gsc* expression (early gastrula) and for visible induction of secondary axes (late neurulas).

### Embryo sections

For vibratome sections, embryos were embedded in a glutaraldehyde-crosslinked gelatin-albumin mix and razor-blade sectioned. Hoechst stained vibratome sections were incubated on microscope slide before imaging (1:10,000 Hoechst). Bisections of embryos was performed using a razor blade.

### Immunofluorescence analysis

Co-injected fluorescein dextran (70,000 MW, ThermoFisher, D1822) was used as lineage tracer for dorsal lips in IF analyses. For IF analyses, embryos were fixed in 4% paraformaldehyde for 1 h at RT, followed by two washes in 1× PBS for 15 min each. For staining of animal caps or bisected specimens, embryos were manually dissected horizontally or sagittaly after fixation, transferred to 24-well plates, and washed twice for 15 min in PBST (PBS/0.1% Triton X-100). After blocking for 2 h at RT in CAS-Block (1:10 in PBST; ThermoFisher, #008120) blocking reagent was replaced by antibody solution (diluted in CAS-Block) for incubation ON at 4°C. Antibodies used were: β1-Integrin (DSHB 8C8-s; 1:70), MZ15 (DSHB; 1:200). Then antibody solution was removed and explants washed twice for 15 min in PBS. Secondary antibodies (ThermoFisher, all 1:1000 in CAS-Block) were incubated for 2 h at RT. Cell borders were visualized using AlexaFluor^TM^Plus 405 Phalloidin (ThermoFisher A30104) overnight at 4°C (1:100 in PBS-). For photo documentation, bisected embryos or caps were transferred onto microscope slides or positioned in low melt agarose on a Petri dish (0.5% low melt agarose in 1× PBS-).

### Photo documentation

LSM images of IF data were taken with a Zeiss LSM 700 Axioplan2 Imaging microscope and then adjusted using the Zeiss Zen 2012 Blue edition. All other pictures were taken with a Zeiss SteREO Discovery. V12 microscope or an Axioplan2 inverted microscope using AxioVision 4.6. Afterwards Adobe Photoshop CS6 was used for cropping and careful brightness adjustments. All figures were arranged using Adobe Illustrator CS6.

### Statistical analysis

Statistical calculations of marker gene expression patterns were performed using Pearson's chi-square test (Bonferroni corrected, if required). *=*P*<0.05, **=*P*<0.01, ***=*P*<0.001 were used for all statistical analyses, as well as the declaration *N*=the number of experiments (i.e. number of biological replicates of batches of embryos from different fertilizations), and *n*=the number of embryos analyzed (i.e. number of biological replicates of embryos).

## Supplementary Material

Supplementary information

## References

[BIO056887C1] Arjonen, A., Alanko, J., Veltel, S. and Ivaska, J. (2012). Distinct recycling of active and inactive β1 integrins. *Traffic* 13, 610-625. 10.1111/j.1600-0854.2012.01327.x 22222055 PMC3531618

[BIO056887C2] Beyer, T., Danilchik, M., Thumberger, T., Vick, P., Tisler, M., Schneider, I., Bogusch, S., Andre, P., Ulmer, B., Walentek, P. et al. (2012). Serotonin signaling is required for Wnt-dependent GRP specification and leftward flow in Xenopus. *Curr. Biol.* 22, 33-39. 10.1016/j.cub.2011.11.027 22177902

[BIO056887C3] Biechele, T. L., Adams, A. M. and Moon, R. T. (2009). Transcription-based reporters of Wnt/beta-catenin signaling. *Cold Spring Harb. Protoc.* 2009, pdb.prot5223. 10.1101/pdb.prot5223 20147181

[BIO056887C4] Bishop, N. and Woodman, P. (2000). ATPase-defective mammalian VPS4 localizes to aberrant endosomes and impairs cholesterol trafficking. *Mol. Biol. Cell* 11, 227-239. 10.1091/mbc.11.1.227 10637304 PMC14770

[BIO056887C5] Borday, C., Parain, K., Thi Tran, H., Vleminckx, K., Perron, M. and Monsoro-Burq, A. H. (2018). An atlas of Wnt activity during embryogenesis in Xenopus tropicalis. *PLoS ONE* 13, e0193606. 10.1371/journal.pone.0193606 29672592 PMC5908154

[BIO056887C6] Bruce, A. E. E. and Winklbauer, R. (2020). Brachyury in the gastrula of basal vertebrates. *Mech. Dev.* 163, 103625. 10.1016/j.mod.2020.103625 32526279

[BIO056887C7] Brunt, L. and Scholpp, S. (2018). The function of endocytosis in Wnt signaling. *Cell. Mol. Life Sci*. 75, 785-795. 10.1007/s00018-017-2654-2 28913633 PMC5809524

[BIO056887C8] Burstyn-Cohen, T., Stanleigh, J., Sela-Donenfeld, D. and Kalcheim, C. (2004). Canonical Wnt activity regulates trunk neural crest delamination linking BMP/noggin signaling with G1/S transition. *Development* 131, 5327-5339. 10.1242/dev.01424 15456730

[BIO056887C9] Butler, M. T. and Wallingford, J. B. (2017). Planar cell polarity in development and disease. *Nat. Rev. Mol. Cell Biol.* 18, 375-388. 10.1038/nrm.2017.11 28293032 PMC5826606

[BIO056887C10] Clevers, H. and Nusse, R. (2012). Wnt/β-catenin signaling and disease. *Cell* 149, 1192-1205. 10.1016/j.cell.2012.05.012 22682243

[BIO056887C11] De Robertis, E. M. (2009). Spemann's organizer and the self-regulation of embryonic fields. *Mech. Dev.* 126, 925-941. 10.1016/j.mod.2009.08.004 19733655 PMC2803698

[BIO056887C12] Dessaud, E., McMahon, A. P. and Briscoe, J. (2008). Pattern formation in the vertebrate neural tube: a sonic hedgehog morphogen-regulated transcriptional network. *Development* 135, 2489-2503. 10.1242/dev.009324 18621990

[BIO056887C13] Dobrowolski, R. and De Robertis, E. M. (2011). Endocytic control of growth factor signalling: multivesicular bodies as signalling organelles. *Nature Publishing Group* 13, 53-60. 10.1038/nrm3244 PMC3374592 22108513

[BIO056887C14] Fagotto, F. (2020). Tissue segregation in the early vertebrate embryo. *Semin. Cell Dev. Biol.* 107, 130-146. 10.1016/j.semcdb.2020.05.020 32600961

[BIO056887C15] Fagotto, F., Guger, K. and Gumbiner, B. M. (1997). Induction of the primary dorsalizing center in Xenopus by the Wnt/GSK/beta-catenin signaling pathway, but not by Vg1, Activin or Noggin. *Development* 124, 453-460. 10.1242/dev.124.2.453 9053321

[BIO056887C16] Fürthauer, M. and González-Gaitán, M. (2009). Endocytic regulation of notch signalling during development. *Traffic* 10, 792-802. 10.1111/j.1600-0854.2009.00914.x 19416471

[BIO056887C17] Glinka, A., Delius, H., Blumenstock, C. and Niehrs, C. (1996). Combinatorial signalling by Xwnt-11 and Xnr3 in the organizer epithelium. *Mech. Dev.* 60, 221-231. 10.1016/S0925-4773(96)00624-7 9025074

[BIO056887C18] Glinka, A., Wu, W., Delius, H., Monaghan, A. P., Blumenstock, C. and Niehrs, C. (1998). Dickkopf-1 is a member of a new family of secreted proteins and functions in head induction. *Nature* 391, 357-362. 10.1038/34848 9450748

[BIO056887C19] Guerra, F. and Bucci, C. (2016). Multiple roles of the small GTPase Rab7. *Cells* 5, 34. 10.3390/cells5030034 PMC5040976 27548222

[BIO056887C20] Hanson, P. I. and Cashikar, A. (2012). Multivesicular body morphogenesis. *Annu. Rev. Cell Dev. Biol*. 28, 337-362. 10.1146/annurev-cellbio-092910-154152 22831642

[BIO056887C21] Heasman, J., Crawford, A., Goldstone, K., Garner-Hamrick, P., Gumbiner, B., McCrea, P., Kintner, C., Noro, C. Y. and Wylie, C. (1994). Overexpression of cadherins and underexpression of beta-catenin inhibit dorsal mesoderm induction in early Xenopus embryos. *Cell* 79, 791-803. 10.1016/0092-8674(94)90069-8 7528101

[BIO056887C22] Heasman, J., Kofron, M. and Wylie, C. (2000). β-catenin signaling activity dissected in the early Xenopus embryo: a novel antisense approach. *Dev. Biol.* 222, 124-134. 10.1006/dbio.2000.9720 10885751

[BIO056887C23] Hikasa, H. and Sokol, S. Y. (2013). Wnt signaling in vertebrate axis specification. *Cold Spring Harbor Perspect. Biol.* 5, a007955-a007955. 10.1101/cshperspect.a007955 PMC3579404 22914799

[BIO056887C24] Honoré, S. M., Aybar, M. J. and Mayor, R. (2003). Sox10 is required for the early development of the prospective neural crest in Xenopus embryos. *Dev. Biol.* 260, 79-96. 10.1016/S0012-1606(03)00247-1 12885557

[BIO056887C25] Hoppler, S. and Moon, R. T. (1998). BMP-2/-4 and Wnt-8 cooperatively pattern the Xenopus mesoderm. *Mech. Dev.* 71, 119-129. 10.1016/S0925-4773(98)00004-5 9507084

[BIO056887C26] Hoppler, S., Brown, J. D. and Moon, R. T. (1996). Expression of a dominant-negative Wnt blocks induction of MyoD in Xenopus embryos. *Genes Dev.* 10, 2805-2817. 10.1101/gad.10.21.2805 8946920

[BIO056887C27] Horner, D. S., Pasini, M. E., Beltrame, M., Mastrodonato, V., Morelli, E. and Vaccari, T. (2018). ESCRT genes and regulation of developmental signaling. *Semin. Cell Dev. Biol.* 74, 29-39. 10.1016/j.semcdb.2017.08.038 28847745

[BIO056887C28] Hounkpe, B. W., Chenou, F., de Lima, F. and De Paula, E. V. (2021). HRT Atlas v1.0 database: redefining human and mouse housekeeping genes and candidate reference transcripts by mining massive RNA-seq datasets. *Nucleic Acids Res.* 49, D947-D955. 10.1093/nar/gkaa609 32663312 PMC7778946

[BIO056887C29] Huotari, J. and Helenius, A. (2011). Focus review endosome maturation. *EMBO J.* 30, 3481-3500. 10.1038/emboj.2011.286 21878991 PMC3181477

[BIO056887C30] Katzmann, D. J., Odorizzi, G. and Emr, S. D. (2002). Receptor downregulation and multivesicular-body sorting. *Nat. Rev. Mol. Cell Biol.* 3, 893-905. 10.1038/nrm973 12461556

[BIO056887C31] Kawamura, N., Sun-Wada, G.-H., Aoyama, M., Harada, A., Takasuga, S., Sasaki, T. and Wada, Y. (2012). Delivery of endosomes to lysosomes via microautophagy in the visceral endoderm of mouse embryos. *Nat. Commun.* 3, 1071-1010. 10.1038/ncomms2069 22990867

[BIO056887C32] Kawamura, N., Takaoka, K., Hamada, H., Hadjantonakis, A.-K., Sun-Wada, G.-H. and Wada, Y. (2020). Rab7-mediated endocytosis establishes patterning of Wnt activity through inactivation of Dkk antagonism. *CellReports* 31, 107733. 10.1016/j.celrep.2020.107733 PMC8171381 32521258

[BIO056887C33] Kim, K., Lake, B. B., Haremaki, T., Weinstein, D. C. and Sokol, S. Y. (2012). Rab11 regulates planar polarity and migratory behavior of multiciliated cells in Xenopus embryonic epidermis. *Dev. Dyn*. 241, 1385-1395. 10.1002/dvdy.23826 22778024 PMC4009926

[BIO056887C34] Kjolby, R. A. S. and Harland, R. M. (2017). Genome-wide identification of Wnt/β-catenin transcriptional targets during Xenopus gastrulation. *Dev. Biol.* 426, 165-175. 10.1016/j.ydbio.2016.03.021 27091726 PMC6288011

[BIO056887C35] Lee, J.-Y. and Harland, R. M. (2010). Endocytosis is required for efficient apical constriction during xenopus gastrulation. *Curr. Biol.* 20, 253-258. 10.1016/j.cub.2009.12.021 20096583 PMC3310928

[BIO056887C36] Li, V. S. W., Ng, S. S., Boersema, P. J., Low, T. Y., Karthaus, W. R., Gerlach, J. P., Mohammed, S., Heck, A. J. R., Maurice, M. M., Mahmoudi, T. et al. (2012). Wnt signaling through inhibition of β-catenin degradation in an intact Axin1 complex. *Cell* 149, 1245-1256. 10.1016/j.cell.2012.05.002 22682247

[BIO056887C37] Margiotta, A., Progida, C., Bakke, O. and Bucci, C. (2017). Rab7a regulates cell migration through Rac1 and vimentin. *BBA Mol. Cell Res.* 1864, 367-381. 10.1016/j.bbamcr.2016.11.020 27888097

[BIO056887C38] Niehrs, C. and Acebron, S. P. (2010). Wnt signaling: multivesicular bodies hold GSK3 captive. *Cell* 143, 1044-1046. 10.1016/j.cell.2010.12.003 21183070

[BIO056887C39] Niehrs, C. and Pollet, N. (1999). Synexpression groups in eukaryotes. *Nature* 402, 483-487. 10.1038/990025 10591207

[BIO056887C40] Nieuwkoop, P. D. and Faber, J. (1994). *Normal Table of Xenopus laevis*. New York: Garland.

[BIO056887C41] Parr, B. A. and McMahon, A. P. (1995). Dorsalizing signal Wnt-7a required for normal polarity of D–V and A–P axes of mouse limb. *Nature* 374, 350-353. 10.1038/374350a0 7885472

[BIO056887C42] Peshkin, L., Lukyanov, A., Kalocsay, M., Gage, R. M., Wang, D., Pells, T. J., Karimi, K., Vize, P. D., Wühr, M. and Kirschner, M. W. (2019). The protein repertoire in early vertebrate embryogenesis. *Preprint, bioRxiv* 1865, 571174. 10.1101/571174

[BIO056887C43] Pla, P. and Monsoro-Burq, A. H. (2018). The neural border: Induction, specification and maturation of the territory that generates neural crest cells. *Dev. Biol.* 444 Suppl. 1, S36-S46. 10.1016/j.ydbio.2018.05.018 29852131

[BIO056887C44] Platta, H. W. and Stenmark, H. (2011). Endocytosis and signaling. *Curr. Opin. Cell Biol.* 23, 393-403. 10.1016/j.ceb.2011.03.008 21474295

[BIO056887C45] Ploper, D. and De Robertis, E. M. (2015). The MITF family of transcription factors: Role in endolysosomal biogenesis, Wnt signaling, and oncogenesis. *Pharmacol. Res*. 99, 36-43. 10.1016/j.phrs.2015.04.006 26003288

[BIO056887C46] Ploper, D., Taelman, V. F., Robert, L., Perez, B. S., Titz, B., Chen, H.-W., Graeber, T. G., von Euw, E., Ribas, A. and De Robertis, E. M. (2015). MITF drives endolysosomal biogenesis and potentiates Wnt signaling in melanoma cells. *Proc. Natl. Acad. Sci. USA* 112, E420-E429. 10.1073/pnas.1424576112 25605940 PMC4321275

[BIO056887C47] Sardiello, M., Palmieri, M., di Ronza, A., Medina, D. L., Valenza, M., Gennarino, V. A., Di Malta, C., Donaudy, F., Embrione, V., Polishchuk, R. S. et al. (2009). A gene network regulating lysosomal biogenesis and function. *Science* 325, 473-477. 10.1126/science.1174447 19556463

[BIO056887C48] Scherz, P. J., Harfe, B. D., McMahon, A. P. and Tabin, C. J. (2004). The limb bud Shh-Fgf feedback loop is terminated by expansion of former ZPA cells. *Science* 305, 396-399. 10.1126/science.1096966 15256670

[BIO056887C49] Schneider, I., Kreis, J., Schweickert, A., Blum, M. and Vick, P. (2019). A dual function of FGF signaling in Xenopus left-right axis formation. *Development* 146, dev173575. 10.1242/dev.173575 31036544

[BIO056887C50] Schulte-Merker, S. and Smith, J. C. (1995). Mesoderm formation in response to Brachyury requires FGF signalling. *Curr. Biol.* 5, 62-67. 10.1016/S0960-9822(95)00017-0 7535172

[BIO056887C51] Session, A. M., Uno, Y., Kwon, T., Chapman, J. A., Toyoda, A., Takahashi, S., Fukui, A., Hikosaka, A., Suzuki, A., Kondo, M. et al. (2016). Genome evolution in the allotetraploid frog Xenopus laevis. *Nature* 538, 336-343. 10.1038/nature19840 27762356 PMC5313049

[BIO056887C52] Shi, D.-L., Bourdelas, A., Umbhauer, M. and Boucaut, J.-C. (2002). Zygotic Wnt/beta-catenin signaling preferentially regulates the expression of Myf5 gene in the mesoderm of Xenopus. *Dev. Biol.* 245, 124-135. 10.1006/dbio.2002.0633 11969260

[BIO056887C53] Sigismund, S., Confalonieri, S., Ciliberto, A., Polo, S., Scita, G. and Di Fiore, P. P. (2012). Endocytosis and signaling: cell logistics shape the eukaryotic cell plan. *Physiol. Rev.* 92, 273-366. 10.1152/physrev.00005.2011 22298658 PMC5614474

[BIO056887C54] Sive, H. L., Grainger, R. M. and Harland, R. M. (2000). *Early Development of Xenopus Laevis*. CSHL Press.

[BIO056887C55] Smith, W. C. and Harland, R. M. (1991). Injected Xwnt-8 RNA acts early in Xenopus embryos to promote formation of a vegetal dorsalizing center. *Cell* 67, 753-765. 10.1016/0092-8674(91)90070-F 1657405

[BIO056887C56] Smith, W. C., McKendry, R., Ribisi, S. and Harland, R. M. (1995). A nodal-related gene defines a physical and functional domain within the Spemann organizer. *Cell* 82, 37-46. 10.1016/0092-8674(95)90050-0 7606783

[BIO056887C57] Sokol, S., Christian, J. L., Moon, R. T. and Melton, D. A. (1991). Injected Wnt RNA induces a complete body axis in Xenopus embryos. *Cell* 67, 741-752. 10.1016/0092-8674(91)90069-B 1834344

[BIO056887C58] Stenmark, H. (2009). Rab GTPases as coordinators of vesicle traffic. *Nature Publishing Group* 10, 513-525. 10.1038/nrm2728 19603039

[BIO056887C59] Stubbs, J. L., Oishi, I., Izpisúa-Belmonte, J. C. and Kintner, C. (2008). The forkhead protein Foxj1 specifies node-like cilia in Xenopus and zebrafish embryos. *Nat. Genet.* 40, 1454-1460. 10.1038/ng.267 19011629 PMC4648715

[BIO056887C60] Tada, M. and Smith, J. C. (2000). Xwnt11 is a target of Xenopus Brachyury: regulation of gastrulation movements via Dishevelled, but not through the canonical Wnt pathway. *Development* 127, 2227-2238. 10.1242/dev.127.10.2227 10769246

[BIO056887C61] Taelman, V. F., Dobrowolski, R., Plouhinec, J.-L., Fuentealba, L. C., Vorwald, P. P., Gumper, I., Sabatini, D. D. and De Robertis, E. M. (2010). Wnt signaling requires sequestration of glycogen synthase kinase 3 inside multivesicular endosomes. *Cell* 143, 1136-1148. 10.1016/j.cell.2010.11.034 21183076 PMC3022472

[BIO056887C62] Teis, D., Wunderlich, W. and Huber, L. A. (2002). Localization of the MP1-MAPK scaffold complex to endosomes is mediated by p14 and required for signal transduction. *Dev. Cell* 3, 803-814. 10.1016/S1534-5807(02)00364-7 12479806

[BIO056887C63] Vick, P., Kreis, J., Schneider, I., Tingler, M., Getwan, M., Thumberger, T., Beyer, T., Schweickert, A. and Blum, M. (2018). An early function of polycystin-2 for left-right organizer induction in Xenopus. *iScience* 2, 76-85. 10.1016/j.isci.2018.03.011 30428378 PMC6136938

[BIO056887C64] Villanueva, S., Glavic, A., Ruiz, P. and Mayor, R. (2002). Posteriorization by FGF, Wnt, and retinoic acid is required for neural crest induction. *Dev. Biol.* 241, 289-301. 10.1006/dbio.2001.0485 11784112

[BIO056887C65] Vinyoles, M., Del Valle-Pérez, B., Curto, J., Viñas-Castells, R., Alba-Castellón, L., García de Herreros, A. and and Duñach, M. (2014). Multivesicular GSK3 sequestration upon Wnt signaling is controlled by p120-catenin/cadherin interaction with LRP5/6. *Mol. Cell* 53, 444-457. 10.1016/j.molcel.2013.12.010 24412065

[BIO056887C66] Vogel, A., Rodriguez, C. and Izpisúa Belmonte, J. C. (1996). Involvement of FGF-8 in initiation, outgrowth and patterning of the vertebrate limb. *Development* 122, 1737-1750. 10.1242/dev.122.6.1737 8674413

[BIO056887C67] Vonica, A. and Gumbiner, B. M. (2002). Zygotic Wnt activity is required for Brachyury expression in the early Xenopus laevis embryo. *Dev. Biol.* 250, 112-127. 10.1006/dbio.2002.0786 12297100

[BIO056887C68] Walentek, P., Schneider, I., Schweickert, A. and Blum, M. (2013). Wnt11b is involved in cilia-mediated symmetry breakage during Xenopus left-right development. *PLoS ONE* 8, e73646-e9. 10.1371/journal.pone.0073646 24058481 PMC3772795

[BIO056887C69] Wessely, O. and Tran, U. (2011). Xenopus pronephros development--past, present, and future. *Pediatr. Nephrol.* 26, 1545-1551. 10.1007/s00467-011-1881-2 21499947 PMC3425949

[BIO056887C70] Yang, Y. and Niswander, L. (1995). Interaction between the signaling molecules WNT7a and SHH during vertebrate limb development: dorsal signals regulate anteroposterior patterning. *Cell* 80, 939-947 10.1016/0092-8674(95)90297-X 7697724

[BIO056887C71] Yokota, C., Mukasa, T., Higashi, M., Odaka, A., Muroya, K., Uchiyama, H., Eto, Y., Asashima, M. and Momoi, T. (1995). Activin induces the expression of the Xenopus homolog of sonic hedgehog during mesoderm formation in Xenopus explants. *Biochem. Biophys. Res. Commun*. 207, 1-7. 10.1006/bbrc.1995.1144 7857250

[BIO056887C72] Yokota, C., Kofron, M., Zuck, M., Houston, D. W., Isaacs, H., Asashima, M., Wylie, C. C. and Heasman, J. (2003). A novel role for a nodal-related protein; Xnr3 regulates convergent extension movements via the FGF receptor. *Development* 130, 2199-2212. 10.1242/dev.00434 12668633

[BIO056887C73] Zhang, B., Tran, U. and Wessely, O. (2011). Expression of Wnt signaling components during Xenopus pronephros development. *PLoS ONE* 6, e26533. 10.1371/journal.pone.0026533 22028899 PMC3197532

