# Supplementary material for: Rab7 is required for mesoderm patterning and gastrulation in Xenopus
Source: Biol Open. 2021 Jul 14;10(7):bio056887. doi: 10.1242/bio.056887 (PMC8325926; doi:10.1242/bio.056887)
Supplement: Supplementary information [file biolopen-10-056887-s1.pdf]

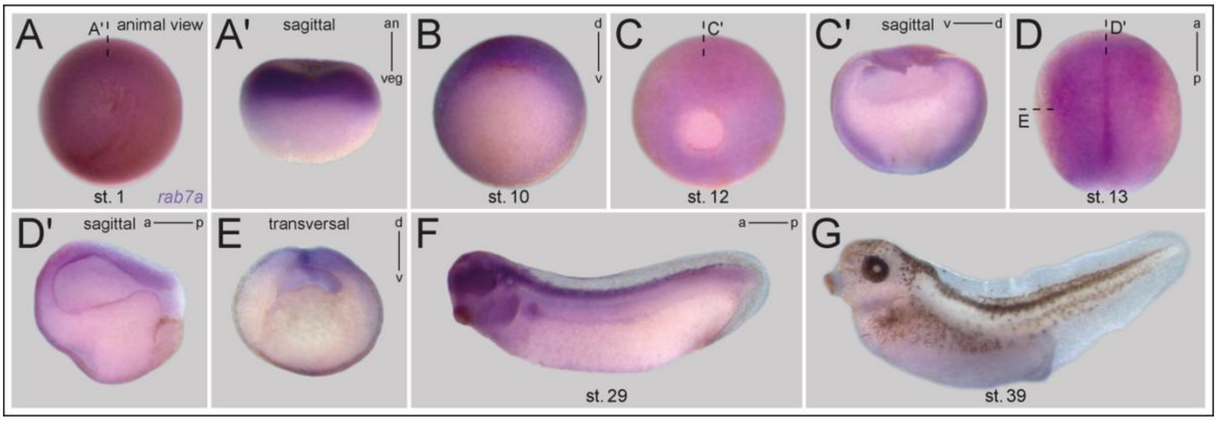


**Fig. S1. *rab7* exhibits dynamic expression pattern.**

(A) *rab7* expression in animal hemisphere of st. 1 embryo, (A') sagittal section of (A). (B,C) Early and late gastrula stages displayed transcripts in deep mesoderm, (C') sagittal section of (C). (D) Onset of neurulation with *rab7* restricted to neural plate and notochord, (D') sagittal section of (D), (E) transversal section as indicated in (D). (F,G) Tailbud stage expression in the notochord, trunk neural crest, eyes, pronephric and head tissues and in dorsal fin mesenchyme.

a, anterior; an, animal; d, dorsal; p, posterior; st., stage; v, ventral; veg, vegetal.

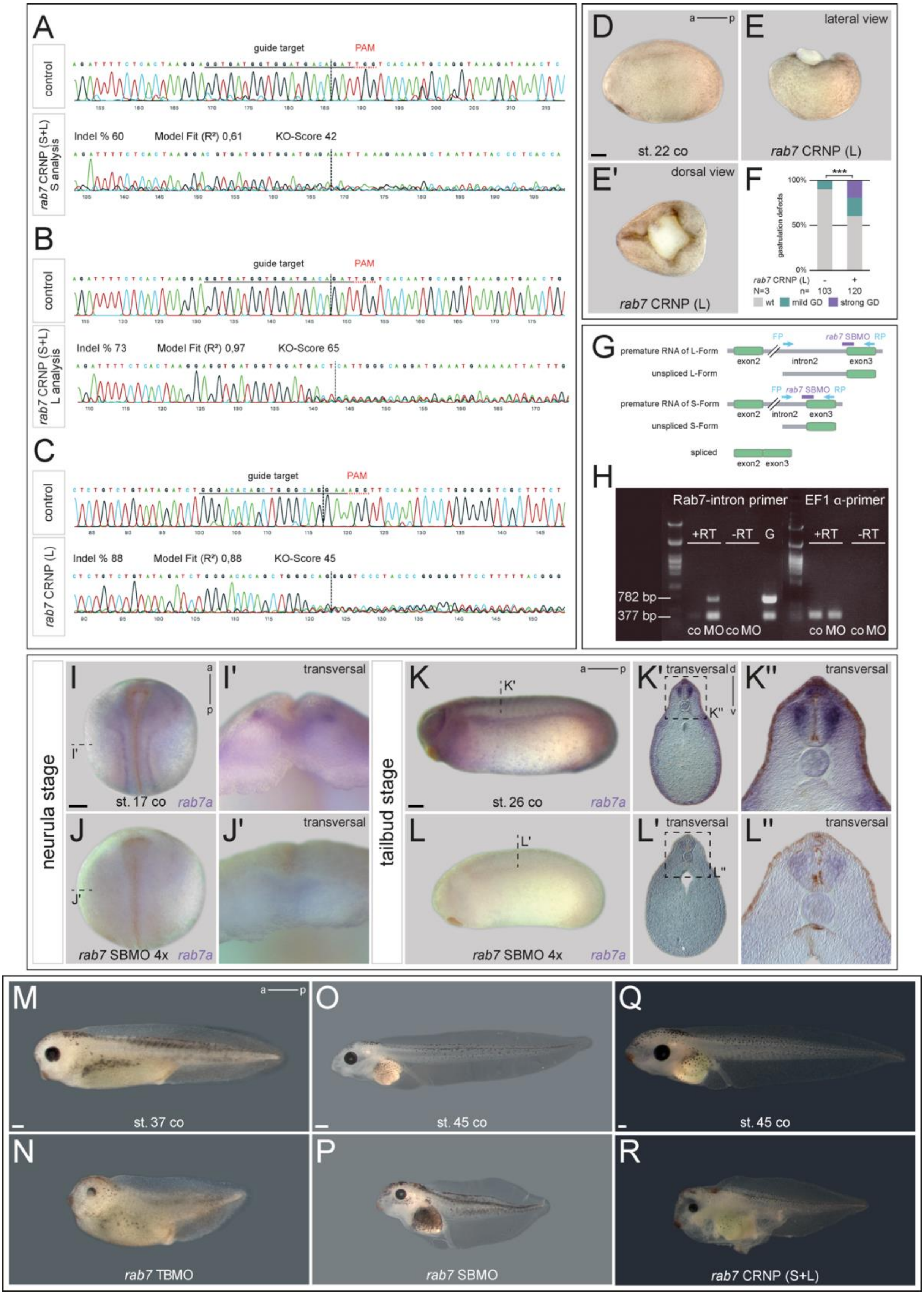


**Fig. S2. Loss of *rab7* caused gastrulation and axis elongation defects.**

(A-C) Synthego ICE CRISPR analysis of *rab7* sgRNAs targeting both S and L alleles or L alone. (D) Wildtype control embryo compared to lateral view of rab7 CRNP (E) treated specimen with severe gastrulation defects, (E') dorsal view. (F) Quantification of results in (D-E). (G) Scheme of binding sites of *rab7* SBMO targeting both homeologs. (H) RT-PCR of knockdown by *rab7* SBMO

Biology Open • Supplementary information

demonstrated successful inhibition of splicing. (I,K) Wildtype neurula and tailbud control specimen with stage specific *rab7* transcripts, (I',K') transversal sections of (I,K), (K'') blow-up of (K'). (J,L) Radial knockdown of *rab7* by SBMO caused significant reduction of *rab7* expression in neurula and tailbud stages, respectively, (J',L') transversal sections of (J,L), (L'') blow-up of (L'). (M,O,Q) Untreated control tadpoles and (N,P,R) milder affected morphant embryos, suffering from AP-axis defects, knockdown was induced using (N) *rab7* TBMO, (P) *rab7* SBMO and (R) *rab7* CRNP (S+L). Scale bars: 250µm

a, anterior; an, animal; co, control; CRNP, Cas9 Ribonucleoprotein; d, dorsal; EF1 α, Elongation factor 1 alpha; FP, forward primer; G, genomic DNA; GD, gastrulation defect; p, posterior; RP, reverse primer; RT, reverse transcriptase; st., stage; v, ventral; veg, vegetal; wt, wildtype.

Biology Open • Supplementary information

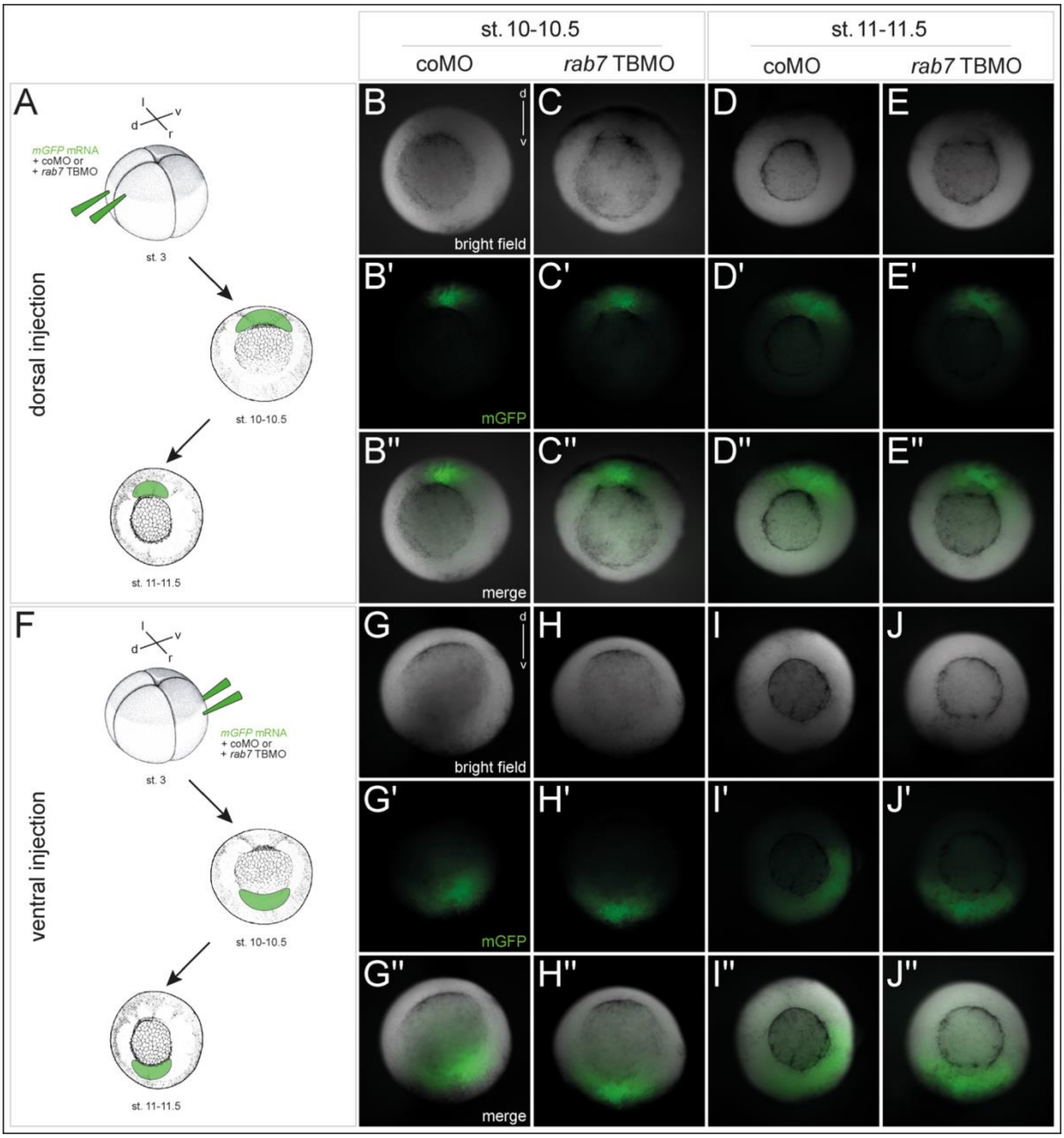


**Fig. S3. Lineage-specific knockdown of *rab7* in the dorsal or ventral mesoderm.**
(A,F) Lineage tracing scheme of injection site (dorsal or ventral) in st. 3 embryos; tested st. 10-10.5 illustrates observed localization of injected material at correct target side (dorsal or ventral), and approximately one stage later before fixation (for late gastrula analyses). *mGFP* mRNA lineage tracer was co-injected with coMO (B,D,G,I) or *rab7* TBMO (C,E,H,J), correct targeting and corresponding phenotypes visualized via fluorescent mGFP (B'-E',G'-J'), brightfield (B-E,G-J) and merged channels (B''-E'',G''-J''). coMO injected specimen depicted normal gastrulation at st. 10-10.5 and 11-11.5, both, when injected dorsally (B,D) or ventrally (G,I). (C,E) Impaired dorsal lip formation of *rab7* morphant embryos at onset of gastrulation, with increasing severeness during gastrulation. (H) Ventral injected specimen did not show altered tissue at st. 10-10.5, (J) impaired lip formation at later gastrula stages with diffuse appearance of ventral lip.
coMO, control Morpholino Oligonucleotide; d, dorsal; p, posterior; st., stage; TBMO, translation blocking Morpholino Oligonucleotide; v, ventral; veg, vegetal.

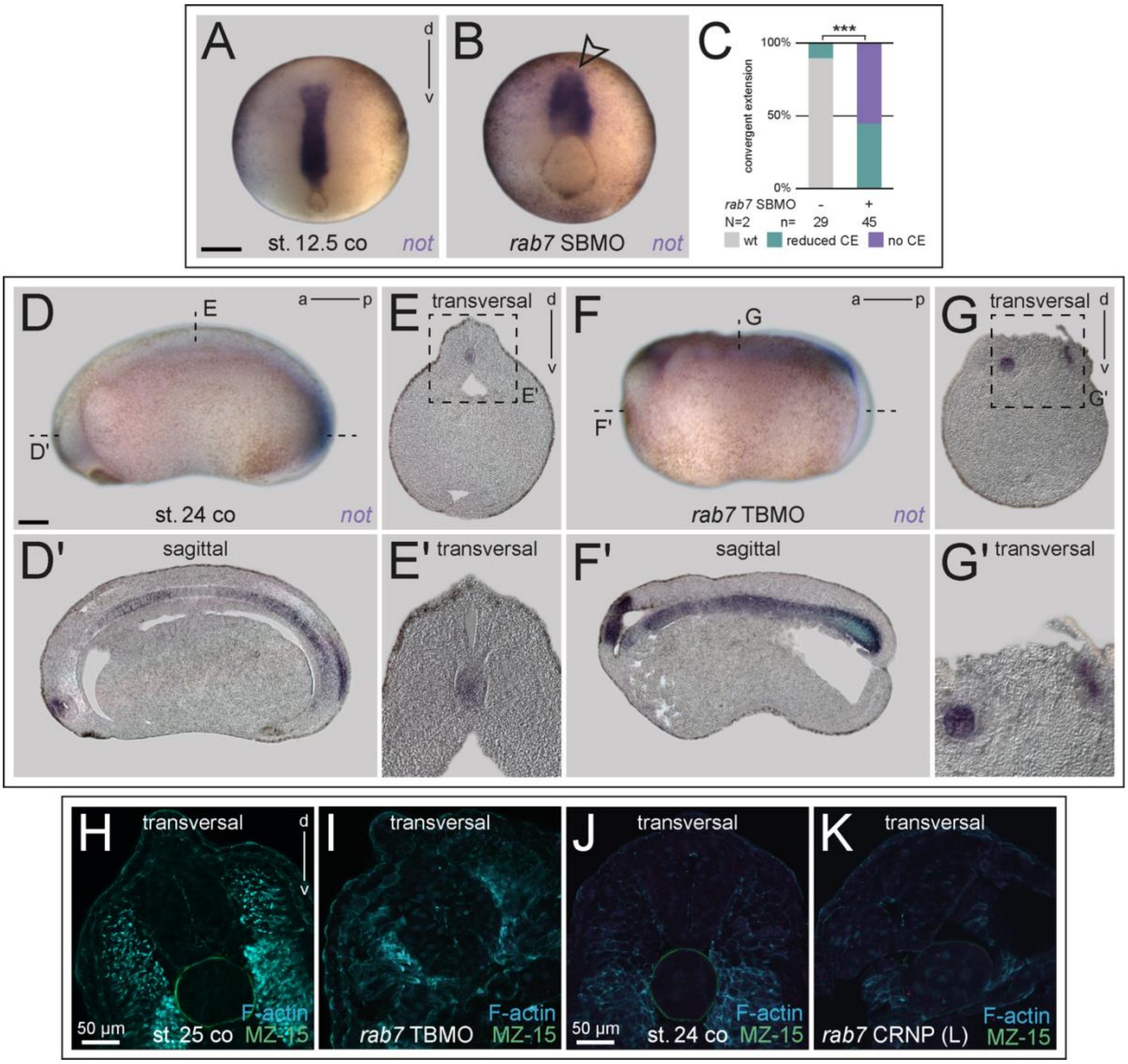


**Fig. S4. *rab7*-deficient embryos develop axial elongation defects and impaired notochord morphogenesis.**

(A) Elongated notochords of control embryos highlighted by *not* expression (B) in comparison to dorsal *rab7* SBMO injection, which resulted in reduced notochord elongation (arrowhead). (C) Quantification of results in (A-B). (D) Tailbud stage embryos revealed *not* expression throughout the notochord, (D') also shown in sagittal and (E) transversal section and (E') blow-up of (E). (F) *rab7* knockdown in dorsal lineage did not reduce *not* expression, (F') but affected notochord morphogenesis depicted in thicker notochord as shown in sagittal section. (G) Transversal sections revealed some morphant specimen also developed split notochords, (G') blow-up highlighting open dorsal tissue. (H-K) Transversal sections of st. 24/25 embryos stained for F-Actin (blue) and MZ-15 (green). (H,J) control specimen displayed wildtype outer sheets of notochords (green), (I) MZ-15 staining was absent in *rab7* TBMO injected or (K) reduced in *rab7* CRNP treated embryos.

Scale bars: (A-G) 250µm; (H-K) 50µm

a, anterior; CE, convergent extension; co, control; CRNP, Cas9 Ribonucleoprotein; d, dorsal; n.s., not significant; p, posterior; st., stage; v, ventral; wt, wildtype.

Biology Open • Supplementary information

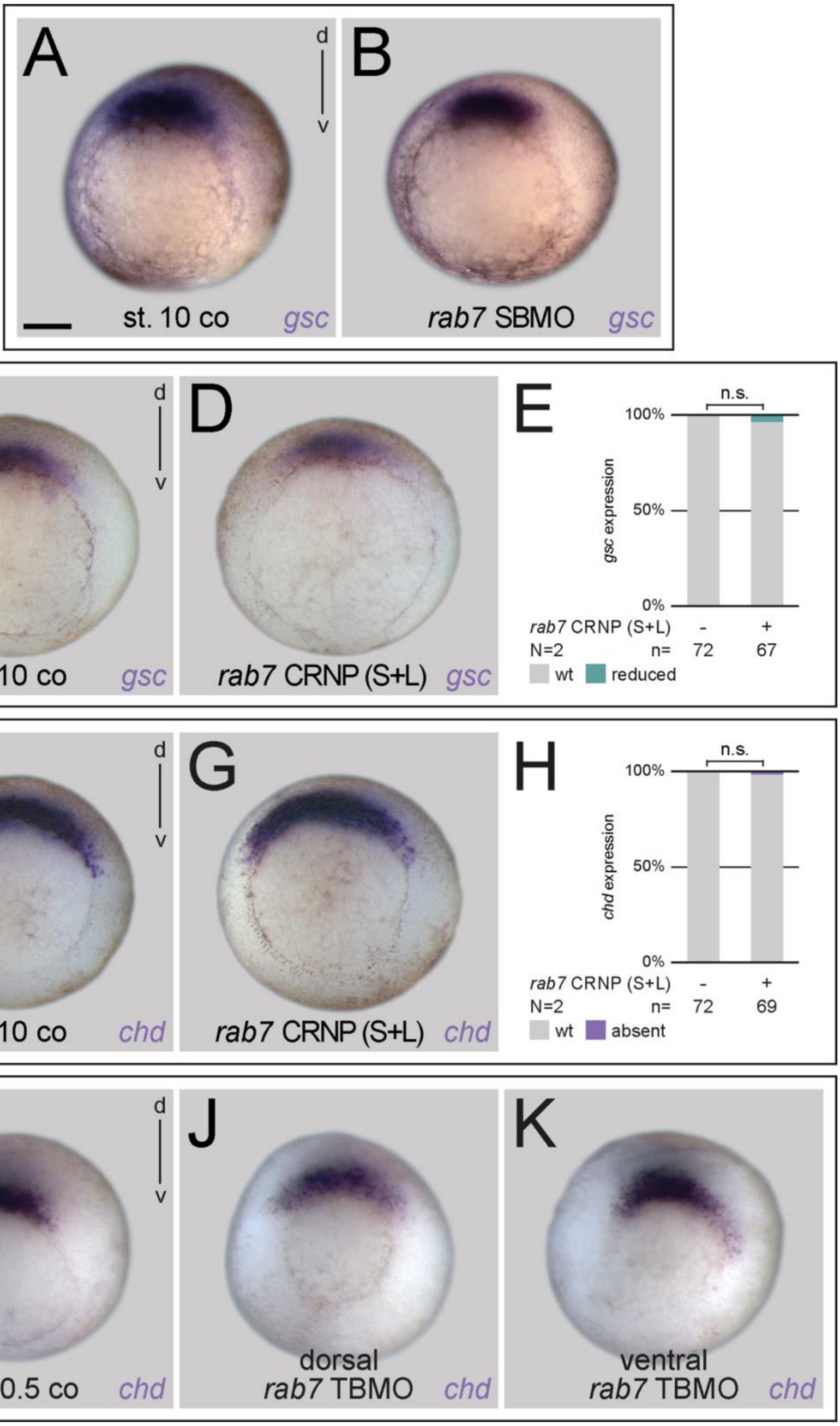


**Fig. S5. Loss of Rab7 does not impact organizer gene expression or DV patterning.**
(A,C) St. 10.5 untreated controls showing wildtype *gsc* expression (B,D) comparable to embryos either injected with *rab7* SBMO or *rab7* CRNP (S+L). (E) Quantification of results in (C-D). (F) Wildtype *chd* expression in control embryos were unaffected in embryos injected with (G) *rab7* CRNP (S+L). (H) Quantification of results in (F-G). (I) DV range of *chd* expression of control embryos was unaltered in embryos injected with *rab7* TBMO in (J) dorsal or (K) ventral lineage.
Scale bars: 250µm
co, control; CRNP, Cas9 Ribonucleoprotein; d, dorsal; n.s., not significant; v, ventral; wt, wildtype.

Biology Open • Supplementary information

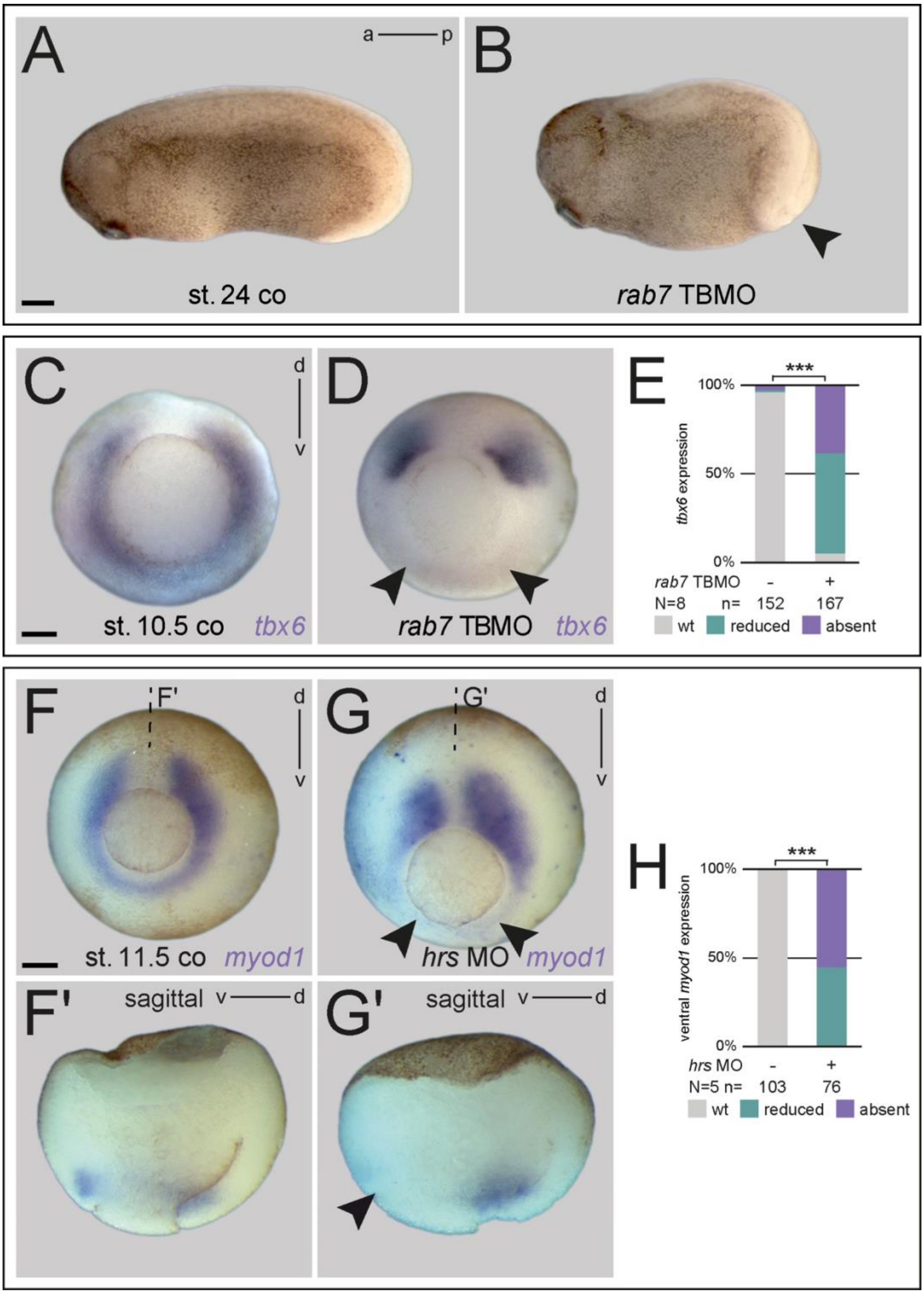


**Fig. S6. A Specification of the ventro-lateral mesoderm requires proper Rab7 function and *hrs* knockdown in the ventral mesoderm phenocopies loss of *rab7*.**
(A) Untreated specimens with normal tail development. (B) Impaired posterior development after loss of *rab7* in ventral lineage (black arrowhead). (C) Control embryo depicting wildtype *tbx6* expression, (D) ventral *rab7* knockdown inhibited *tbx6* expression (black arrowheads). (E) Quantification of results in (C-D). (F) Wildtype *myod1* expression in control specimen, (F') sagittal section of (F) showing ventral expression. (G) Ventral injection of *hrs* MO (0,8-1 pmol) caused loss of *myod1* (black arrowheads), (G') sagittal section as indicated in (G) with absent ventral expression (black arrowhead). (H) Quantification of results. Scale bars: 250µm
a, anterior; co, control; d, dorsal; MO, morpholino oligonucleotide; p, posterior; st., stage; v, ventral; wt, wildtype.

Biology Open • Supplementary information

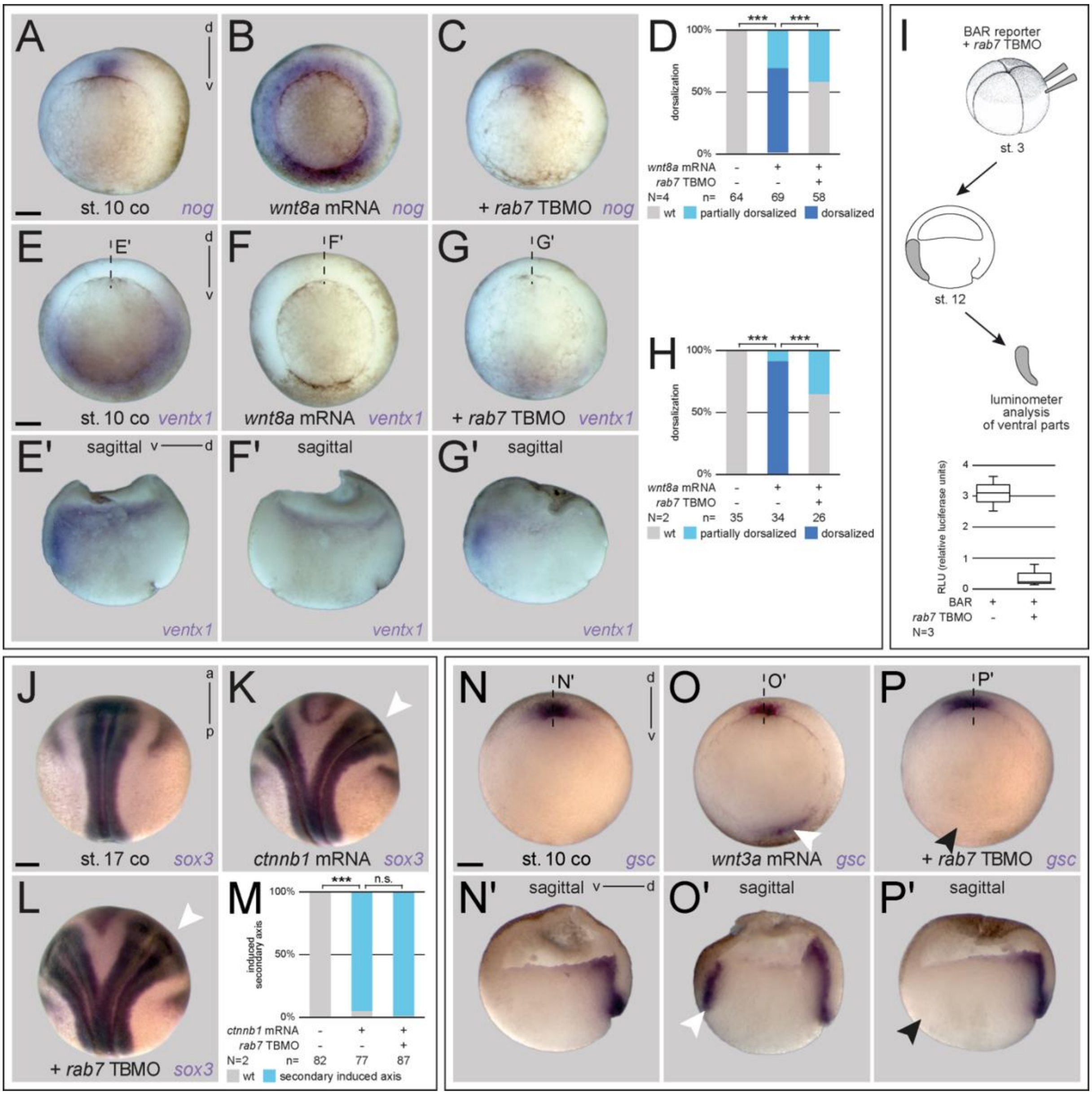


**Fig. S7. Rab7 is required for exogenous activation of canonical Wnt pathway.**

(A) Wildtype dorsal expression of *nog* in st. 10 embryos in comparison to specimen with (B) radial injected *wnt8a* mRNA showing complete dorsalization by extended *nog* expression around the blastopore. (C) Co-injection of *rab7* TBMO restricted *nog* expression to normal wildtype area again. (D) Quantification of results in (A-C). (E) Wildtype ventral expression of *ventx1* in st. 10 embryos, (E') sagittal section as indicated in (E) showing *ventx1* expression domain. (F) Loss of *ventx1* expression upon radial *wnt8a* mRNA injection, (F') highlighted in bisected embryo. (G) Co-injection of *rab7* TBMO partially restored lost *ventx1* expression, (G') which in the sagittal section remained smaller and weaker on ventral side. (H) Quantification of results in (E-G). Please note premature (st. 10) appearance of fully radial lips in (B,F) but not in (A,C,E,G) in same age specimens. (I) Late gastrula Luciferase-based BAR-reporter assay (bottom) demonstrating tissue-specific inhibition of endogenously induced Wnt reporter activity in the ventral mesoderm (middle) after co-injection of *rab7* TBMO into the ventral mesodermal lineage at 4-cell stage (top). (J) Wildtype AP axis in control specimen. (K) *ctnnb1* induced right-sided secondary axis (white arrowhead), (L) which was not inhibited by *rab7* knockdown (white arrowhead). (J-L) Axes highlighted by *sox3* expression. (M) Quantification of results in (J-L). (N) Control embryos showing normal dorsal *gsc* expression, (N') confirmed in sagittal section indicated in (N). (O) Ventral *wnt3a* mRNA injection induced second

organizer marked by *gsc* (white arrowhead), (O') sagittal section revealed additional ventral *gsc* positive domain (white arrowhead). (P) Lost second *gsc* expression upon parallel *rab7* TBMO injection (black arrowhead), (P') highlighted in sagittal section (black arrowhead).
Scale bars: 250µm
a, anterior; co, control; d, dorsal; n.s., not significant; p, posterior; v, ventral; wt, wildtype.
